# *Bacillus subtilis* engineered for topical delivery of an antifungal agent

**DOI:** 10.1371/journal.pone.0293664

**Published:** 2023-11-30

**Authors:** Veronica A. Montgomery, Ethan Cain, Mark P. Styczynski, Mark R. Prausnitz

**Affiliations:** 1 Wallace H. Coulter Department of Biomedical Engineering at Emory University and Georgia Tech, Georgia Institute of Technology, Atlanta, GA, United States of America; 2 School of Chemical & Biomolecular Engineering, Georgia Institute of Technology, Atlanta, GA, United States of America; University of Jeddah, SAUDI ARABIA

## Abstract

Fungal skin infections are a common condition affecting 20–25 percent of the world population. While these conditions are treatable with regular application of an antifungal medication, we sought to develop a more convenient, longer-lasting topical antifungal platform that could increase patient adherence to treatment regimens by using *Bacillus subtilis*, a naturally antifungal bacteria found on the skin, for drug production and delivery. In this study, we engineered *B*. *subtilis* for increased production of the antifungal lipopeptide iturin A by overexpression of the pleiotropic regulator DegQ. The engineered strain had an over 200% increase in iturin A production as detected by HPLC, accompanied by slower growth but the same terminal cell density as determined by absorbance measurements of liquid culture. In an *in vitro* antifungal assay, we found that despite its higher iturin A production, the engineered strain was less effective at reducing the growth of a plug of the pathogenic fungus *Trichophyton mentagrophytes* on an agar plate compared to the parent strain. The reduced efficacy of the engineered strain may be explained by its reduced growth rate, which highlights the need to address trade-offs between titers (e.g. measured drug production) and other figures of merit (e.g. growth rate) during metabolic engineering.

## Introduction

Dermatophytoses are fungal infections of the skin [1]. Common dermatophytoses include tinea pedis (athletes’ foot), tinea corporis (ringworm), tinea capitis (scalp infection), and onychomycosis (nail infection). An estimated 20 to 25 percent of the world’s population is affected by fungal skin infections, and it is estimated that over $500 million is spent each year to treat these conditions [2,3]. Topical application of antifungal agents is the primary form of treatment for tinea pedis and tinea corporis. These treatment regimens involve daily or twice-daily application of an antifungal agent (typically azoles or allylamines) for 1 to 6 weeks [4]. The requirement for frequent application, however, can reduce patient adherence, making a more convenient delivery method desirable. Several studies have sought to address this limitation by developing slow-release antifungal delivery platforms through encapsulation of antifungal drugs in novel materials for enhanced skin penetration and controlled release. Examples include layered double hydroxides, niosomes, and nanostructured lipid carriers [5–7].

We hypothesized that the required frequency of antifungal administration could be further improved by developing a microbiome-based platform to continuously deliver antifungal drugs to the skin surface. *B*. *subtilis* naturally produces antifungal compounds in the iturin, surfactin, and fengycin families. In fact, the commercial agricultural product Serenade uses *B*. *subtilis* to fight plant fungal pathogens, and *B*. *subtilis* has been incorporated into microneedles and a topical gel for treatment of skin fungal infections in mice [8–10]. Further supporting its use as a topical antifungal treatment, *B*. *subtilis* is found within the skin microflora, has Generally Regarded as Safe status from the FDA, and is commercially available for human consumption [11–13]. In this study, we sought to increase the antifungal activity of *B*. *subtilis* by engineering cells to overproduce the antifungal lipopeptide iturin A.

Iturin A is a broad antifungal agent active against several relevant fungi, including *Trichophyton mentagrophytes* and *Trichophyton rubrum*, two of the main causes of athlete’s foot [2,14,15]. While iturin A has not been widely used as a therapeutic due to high production costs, low yields, and hemolytic activity, we hypothesized that *in situ* production of iturin A by *B*. *subtilis* cells localized to the skin surface would eliminate the need for costly extraction procedures and exhibit minimal safety concerns [9,16,17]. We thus expected this strategy to serve as an effective means to reduce the frequency of required antifungal administrations by providing continuous drug exposure.

In this study, we engineered *B*. *subtilis* to have increased production of iturin A using the pleiotropic regulator DegQ, which has been used by others to increase iturin A production in strains of *Bacillus* [18,19]. We then studied the effectiveness of this engineered strain in an *in vitro* antifungal assay.

## Methods

### Media and growth conditions

Bacterial and fungal strains used in this study are listed in Table 1. *Escherichia coli* and *B*. *subtilis* were grown in lysogeny broth (LB), consisting of 0.5% w/v yeast extract (Life Technologies, Carlsbad, CA), 1% w/v NaCl, and 1% w/v tryptone (Life Technologies) at 37°C and shaking at 200 rpm with antibiotics (100 μg/ml carbenicillin and 10 μg/ml kanamycin for *E*. *coli* and *B*. *subtilis*, respectively). Growth of cells was monitored by reading absorbance at 600 nm using a Biotek Synergy H4 Hybrid Microplate Reader (Agilent, Santa Clara, CA).

**Table 1:** Bacteria, fungi, and DNA plasmids.

Name	Description	Source/Reference
**Strains**		
168	*B*. *subtilis* common laboratory strain	ATCC^1^
15841	Antifungal lipopeptide-producing strain of *B*. *subtilis*	ATCC, [20]
BS-DegQ	Strain 15841 harboring pDegQ plasmid	This study
DH5α	*E*. *coli* competent cells	NEB^2^
*T*. *mentagrophytes*	Fungus that causes athlete’s foot	ATCC
**Plasmids**		
pRB374	*E*. *coli*—*B*. *subtilis* shuttle plasmid; amp^r^, kan^r^	ATCC, [21]
pDegQ	vegII:degQ; amp^r^, kan^r^	This study

^1^ ATCC, American Type Culture Collection (Manassas, VA).

^2^ NEB, New England Biolabs (Ipswich, MA).

Landy medium, consisting of 20 g/L glucose, 5 g/L L-glutamic acid, 0.5 g/L MgSO_4_, 0.5 g/L KCl, 1 g/L KH_2_PO_4_, 0.15 mg/L FeSO_4_, 5 mg/L MnSO_4_, and 0.16 mg/L CuSO_4_ and raised to pH 7 with 1 N NaOH, was used for iturin A production [22].

*T*. *mentagrophytes* was grown on Emmons Modified Sabouraud Dextrose Agar (BD Difco, Franklin Lakes, NJ) at 25°C. After a lawn was formed (about 15–20 days), spores were harvested by adding sterile PBS with 0.05% v/v Tween 20 to the plate surface and then gently scraping the surface with a sterile L-shaped cell spreader. After harvesting, the spore suspension was either plated on Sabouraud agar for continued culture or stored in 15% glycerol at -80°C for future use.

### Plasmid assembly

Plasmids used in this study are listed in Table 1. Primers used in this study are listed in S1 Table. All plasmids were assembled in *E*. *coli* DH5α and then transformed into *B*. *subtilis* 15841. *E*. *coli* cells were transformed by heat shock following the NEB High Efficiency Transformation protocol (New England Biolabs, Ipswich, MA). *B*. *subtilis* cells were transformed by electroporation using an ECM 600 electroporator (BTX, Holliston, MA) set to 2.1 kV, 129 Ω and 50 μF with 1 mm gap cuvettes.

Iturin A is a non-ribosomal antifungal lipopeptide that is encoded in the *B*. *subtilis* 15841 genome by a 37.2 kb gene cluster, encompassing genes ituD (1203 bp), ituA (11949 bp), ituB (16089 bp), and ituC (7854 bp). The genome of *B*. *subtilis* 15841 was extracted using the Purelink Genomic DNA Minikit (ThermoFisher, Waltham, MA) and sequenced with the Illumina MiSeq Nano kit (San Diego, CA). The resulting reads were aligned with the published sequence for the iturin A gene cluster in *B*. *subtilis* strain RB14 using the Illumina BWA Aligner online application [23,24]. The annotated sequence of the iturin A gene cluster in strain 15841 is available in S2 Table. Due to the size of the gene cluster, it was amplified from the 15841 genome extract by PCR in 3 parts: ituD/A (13152 bp, primers ituDA 5p.F and ituDA 3p.R), ituB1 (12052 bp, primers ituB1 5p.F and ituB1 3p.R), and ituB/C (12019 bp, primers ituBC 5p.F and ituBC 3p.R).

The degQ gene (WP_003220708.1) was synthesized by Eurofins (Louisville, KY). Plasmid pDegQ for the overexpression of DegQ in *B*. *subtilis* 15841 was created by inserting the degQ gene into the shuttle plasmid pRB374 by Gibson assembly [25].

### Iturin A extraction from liquid culture

Overnight cultures of *B*. *subtilis* strain 15841 or BS-DegQ were diluted in fresh Landy medium and grown for 3 to 7 days at 37°C, shaking at 200 rpm. Cultures were then centrifuged for 15 min at 3000 rcf and 4°C, and iturin A was extracted from culture supernatant using an aqueous two-phase extraction method [26]. To create a turbid solution, 1 mL of culture supernatant was combined with 0.2 mL ethanol. The pH was adjusted to 9 using 1 N NaOH, and 0.5 g (NH_4_)_2_SO_4_ was added. The mixture was vortexed until the salt dissolved completely and then centrifuged for 5 min at 6000 rcf. The upper phase was preserved for analysis, and then the lower phase was extracted again with 0.2 mL ethanol. The upper phases were then combined, diluted to 1 mL with methanol, and then filtered through a 0.2 μm filter for HPLC analysis.

### Analytical methods

Iturin A was detected using an Agilent 1200 series HPLC system (Agilent, Santa Clara, CA) equipped with a reversed-phase HPLC column (Eclipse XDB-C18, 5 μm, 4.6 x 150 mm, Agilent) at a wavelength of 230 nm. The mobile phase consisted of 60% water with 0.1% trifluoroacetic acid and 40% acetonitrile at a flow rate of 0.6 mL/min and a temperature of 20°C. Commercial iturin A was used as a standard (Sigma, St. Louis, MO). Because we observed a peak for Landy medium around one of the expected iturin A peaks, only peaks at 8.1 and 8.6 min were used for calculating iturin A concentration.

### Antifungal assays

An 8-mm biopsy punch was taken from a lawn of *T*. *mentagrophytes* on Sabouraud agar and transferred to a clean Sabouraud agar plate. Overnight cultures of bacteria were diluted in fresh LB media and grown at 37°C and shaking at 200 rpm until they reached early log phase. Cultures were then adjusted to a concentration of 10^6^ CFU/mL and streaked along a line 3 cm away from the fungal plug using a sterile cotton swab (Puritan Medical Products, Guilford, ME). The plates were left at room temperature (20–25˚C) for 15 days and then imaged using a UVP UVsolo touch stand-alone gel documentation system (Analytik Jena, Jena, Germany). Antifungal activity was assessed by observing the growth of the fungal plug.

### Statistical analysis

Results are presented as mean +/- standard deviation. One-way ANOVA followed by Dunnett’s multiple comparisons test was used to compare doubling times, which were calculated by fitting the mid-exponential phase of the growth curves to an exponential growth equation. Statistical analyses were performed using GraphPad Prism version 9.4.1 for Windows (San Diego, California, www.graphpad.com). A p-value less than 0.05 was considered significant.

## Results

**Fig 1:**
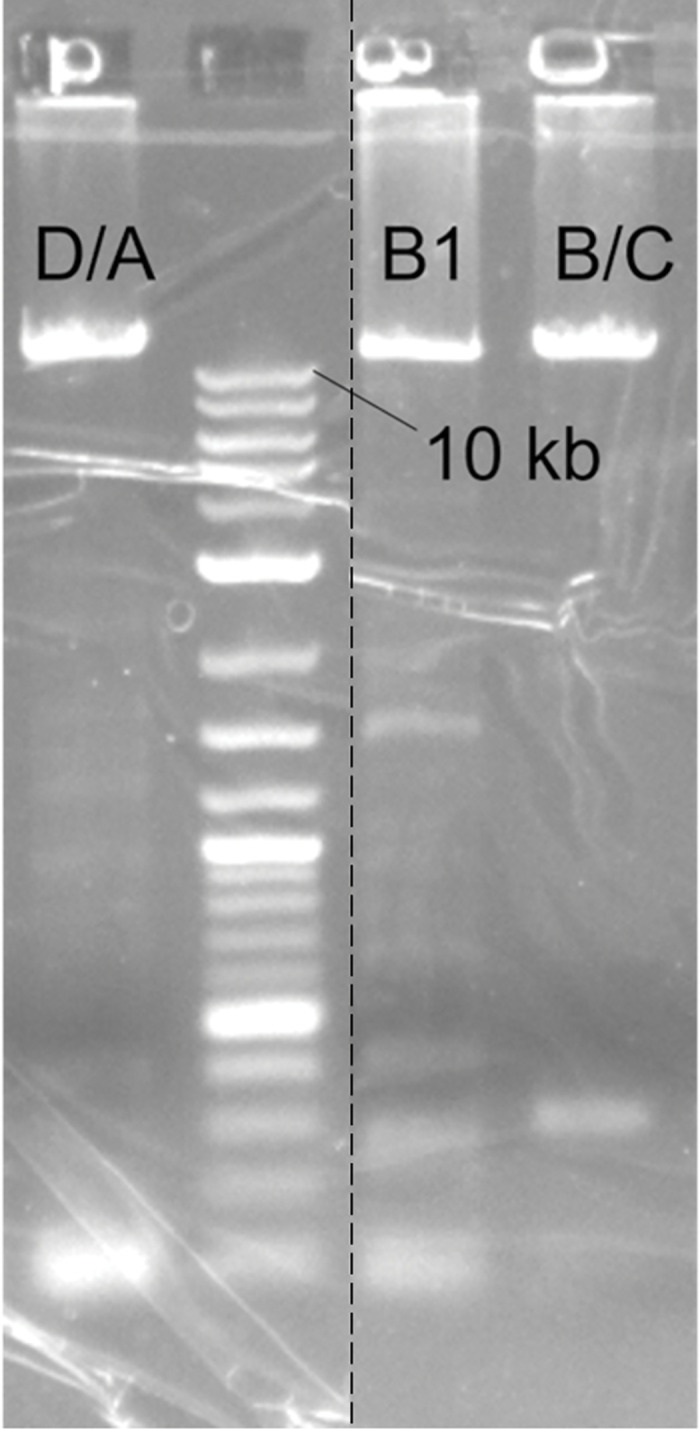
Amplification of iturin A gene cluster from *B*. *subtilis* strain 15841 genomic DNA by PCR. D/A encompasses a region including ituD and ituA. B1 encompasses part of ituB. B/C encompasses the remaining section of ituB and ituC. While individual parts of the cluster were successfully amplified, we were unable to isolate a single insert containing the whole cluster. Image has been cropped to remove duplicate lanes (dashed line). The full image is available in S1 Raw image.

We first confirmed that *B*. *subtilis* strain 15841 has the iturin A gene cluster in its genome by amplifying the entire gene cluster from the genome extract. Due to the size of the gene cluster, it was amplified in three parts (ItuD/A, 13152 bp; ItuB1, 12052 bp; ItuB/C, 12019 bp) (Fig 1). We attempted to combine these parts into a single plasmid for potential plasmid-borne overexpression but were not successful, likely due to an insert of such a large size being better suited for a cosmid or alternative vector [27].

**Fig 2:**
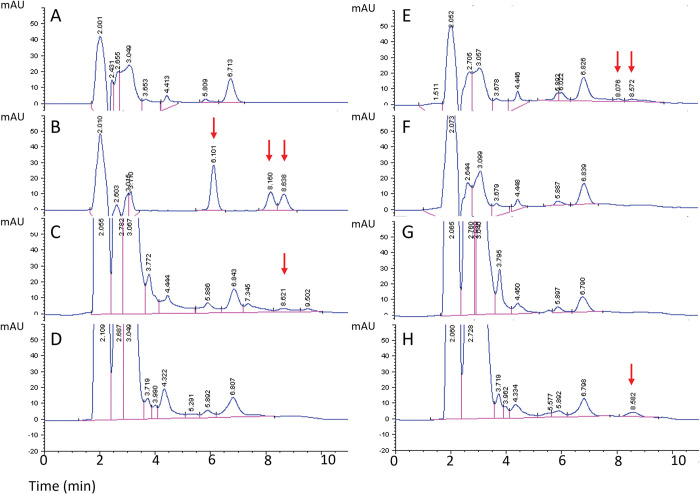
Representative HPLC chromatograms for analysis of iturin A concentration. (A) Landy medium. (B) Iturin A at a concentration of 75 μg/mL in methanol. (C) *B*. *subtilis* strain 15841 cultured for (C) 3 days or (D) 7 days. Landy medium (E) spiked with 10 μg/mL iturin A and (F) 7 days after being spiked with 10 μg/mL iturin A. *B*. *subtilis* engineered to over-express iturin A (BS-DegQ) cultured for (G) 3 days or (H) 7 days. Iturin A slowly accumulates in BS-DegQ (G, H), but also appears to degrade after production or spike-in (C vs. D, E vs. F). Arrows indicate peaks that were considered characteristic of iturin A.

We then engineered *B*. *subtilis* strain 15841 for increased production of iturin A by overexpressing the pleiotropic regulator DegQ on plasmid DNA. DegQ facilitates DegS phosphorylation of DegU, which in turn regulates production of lipopeptides in species of *Bacillus* [28,29]. A prior study showed that heterologous expression of DegQ on plasmid DNA led to higher iturin A production compared to heterologous expression of DegU in *Bacillus amyloliquefaciens* [19], which suggested that overexpression of DegQ might also be a good approach for this study. We tested whether the engineered *B*. *subtilis* strain (BS-DegQ) was successfully engineered to produce higher levels of iturin A by HPLC analysis of the cell culture supernatant. Iturin A contains a cyclic heptapeptide with a β-amino fatty acid chain [30]. There are multiple iturin A homologues corresponding to different carbon chain lengths (primarily 14, 15 and 16 carbons), which can be characterized by three distinct HPLC peaks at 6.1, 8.1 and 8.6 min (Fig 2B) [26].

There was a peak in Landy medium (i.e., the negative control) around one of the expected peaks for iturin A (5.8 min) (Fig 2A), so peaks near that elution time were not considered for calculations of extracted iturin A. Landy medium spiked with 10 μg/mL of commercial iturin A was used as a positive control and exhibited the expected characteristic peaks at 8.1 and 8.6 min (Fig 2E).

*B*. *subtilis* strain 15841, which naturally produces iturin A, was used as another positive control. It produced detectable iturin A in the culture supernatant after 3 days, as evidenced by a peak at 8.6 min (Fig 2C), but no iturin A was detected after 7 days (Fig 2D). Iturin A was also not detectable in Landy medium spiked with 10 μg/mL iturin A after 7 days in culture conditions (Fig 2F), suggesting that iturin A synthesized by cells in Landy medium can only be measured reliably within a few days of its production. BS-DegQ did not show evidence of iturin A production after 3 days (Fig 2G), but a peak corresponding to iturin A at 8.6 min appeared after 7 days of culture (Fig 2H). We believe that only one of the characteristic iturin A peaks was seen in these culture supernatants because other peaks may have been below the limit of detection. Differences in the ratio of iturin A homologues have been observed between different strains of *B*. *subtilis* as well as by the same strain of *B*. *subtilis* grown in different culture conditions [18,31].

We next quantified iturin A concentration in the culture supernatant of the *B*. *subtilis* strains. The iturin A concentration in the culture supernatant of strains 15841 and BS-DegQ after 3 and 7 days of culture, respectively, were of similar magnitude, but iturin A produced by BS-DegQ achieved a concentration approximately three-fold higher than strain 15841 (Student’s t-test, p = 0.0016) (Table 2). Altogether, these data indicate that BS-DegQ, at the time points measured, produced a higher concentration of iturin A than the parent stain 15841.

**Table 2:** Iturin A detected from liquid culture by HPLC.

	Iturin A concentration (μg/mL)^1^	
*B*. *subtilis* strain	Day 3	Day 7
15841 (parent)	2.24 +/- 0.16	0
BS-DegQ	0	7.39 +/- 0.63

^1^Values represent average of 3 replicates +/- standard deviation.

**Fig 3:**
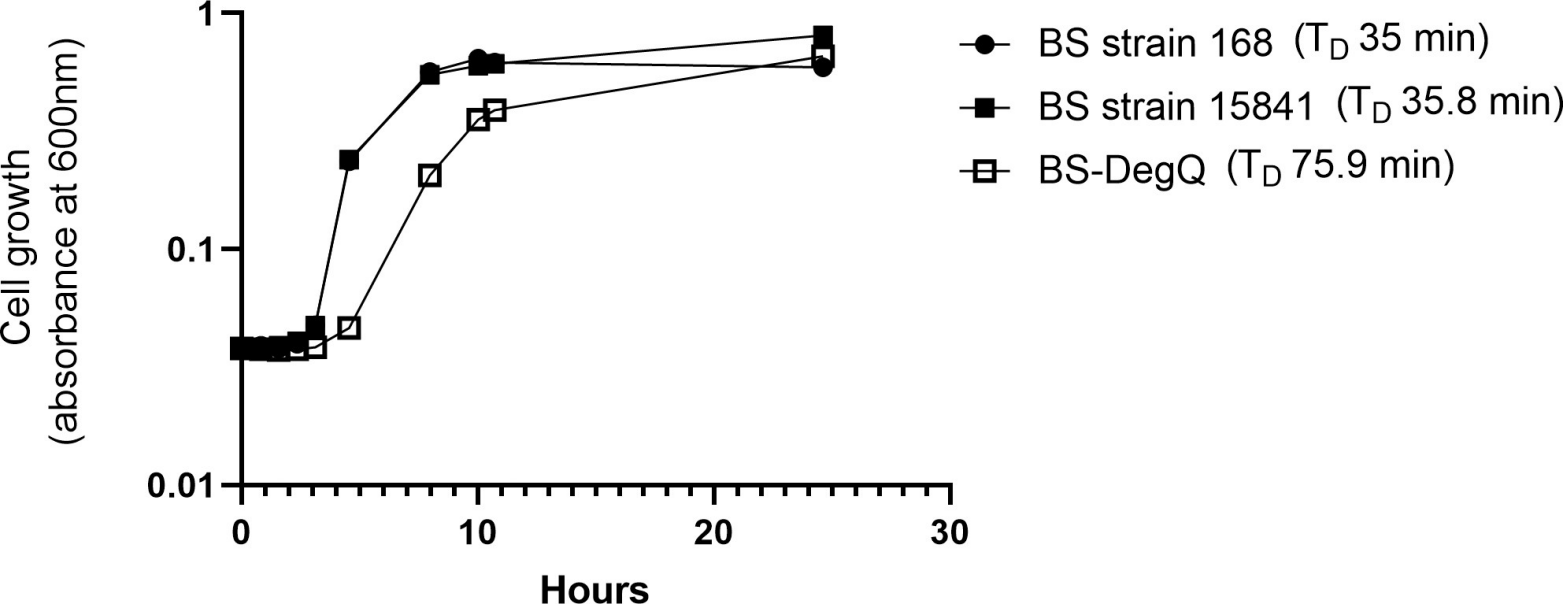
Representative growth curves of *B*. *subtilis* (BS) strains 168, 15841, and BS-DegQ. Cells were cultured for one day in LB media with no antibiotic at 37°C with shaking. The BS-DegQ strain showed a substantial increase in doubling time.

We wanted to better understand the kinetics of iturin A production by the different *B*. *subtilis* strains and thought it might relate to their growth kinetics. The unmodified strains 15841 (parent strain) and 168 (negative control) exhibited similar doubling times of 35.8 +/- 0.9 min (n = 3) and 35.0 +/- 2.1 min (n = 3), respectively (p = 0.995) (Fig 3). In contrast, the engineered BS-DegQ strain had a significantly longer doubling time of 75.9 +/- 17.27 min (n = 3) (p = 0.007 and p = 0.006 for 15841 and 168, respectively). We hypothesize that the longer doubling time of BS-DegQ may explain the delayed appearance of iturin A in the supernatant during liquid culture compared to the parent strain 15841.

**Fig 4:**
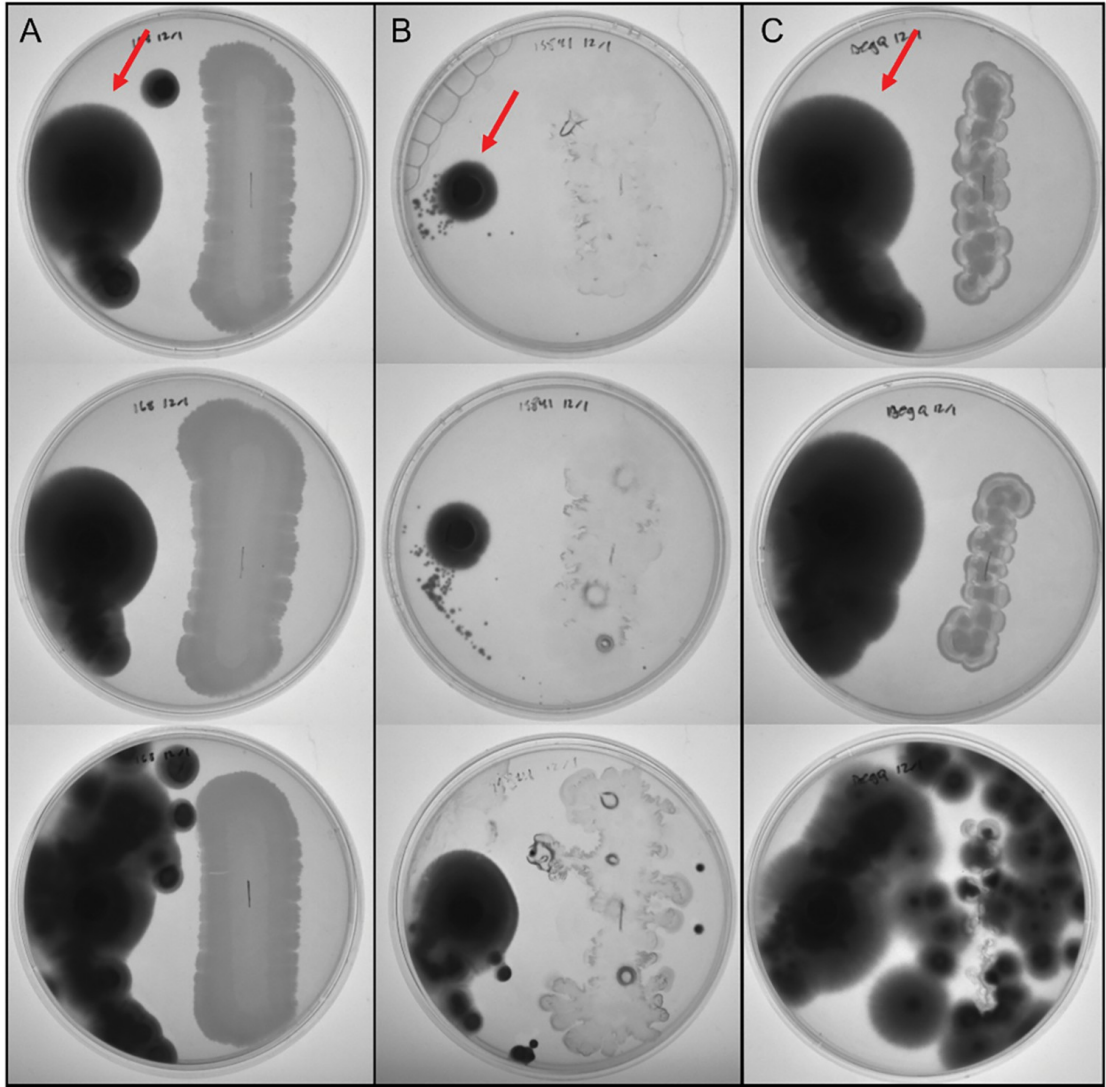
*In vitro* antifungal assay. *T*. *mentagrophytes* (left side of each plate) were cultured with *B*. *subtilis* (A) strain 168, (B) strain 15841 or (C) strain BS-DegQ for 15 days at room temperature. Each column shows three replicates of the same *B*. *subtilis* strain. Arrows in the top row indicate the fungus *T*. *mentagrophytes*. Diffuse growth of 15841 cells may be due to the production of biosurfactants such as surfactin, which facilitate cell sliding on agar plates.

We finally tested the hypothesis that a strain with increased iturin A production (BS-DegQ) would have increased antifungal activity. In an *in vitro* antifungal assay, we compared the ability of different strains to control the growth of a plug of *T*. *mentagrophytes* on an agar plate (Fig 4). In cultures containing the negative control 168 strain (i.e., does not produce iturin A), we saw growth of the fungal cells (appearing black) as well as growth of the 168 cells (appearing gray) (Fig 4A).

In contrast, fungal cells in the presence of the parent strain 15841 exhibited very little growth (Fig 4B), presumably due to the endogenous antifungal activity of these bacterial cells [20]. The 15841 cells appear to be diffuse on the agar plate, possibly because of the production of other biosurfactants such as surfactin. While we did not analyze surfactin production in this study, strain 15841 has also been reported to produce surfactin, and surfactin production facilitates cell sliding on agar plates [32,33].

Finally, we found that the BS-DegQ strain did not appear to inhibit fungal cell growth (Fig 4C). The increased growth of fungus was accompanied by less growth of bacteria for the BS-DegQ strain, which is consistent with the slower growth kinetics seen in Fig 3. Slower growth would lead to increased early fungal proliferation as BS-DegQ grew and began iturin A production. Heterologous DegQ expression has also been found to decrease production of surfactin, another antifungal lipopeptide which we did not analyze in this study but could also contribute to the observed reduction in antifungal activity [28].

## Discussion

While fungal skin infections are generally treatable with topical application of an antifungal medication, the requirement for frequent administrations (1 to 2 times per day) is an inconvenience that can lead to poor adherence by patients. Adherence could potentially be improved by the use of longer-lasting topical delivery platforms that require less frequent application [4].

In this study we explored the possibility of using *B*. *subtilis* as an extended-release topical antifungal treatment. *B*. *subtilis* naturally produces multiple antifungal molecules, including iturin A, an antifungal lipopeptide that is active against key fungi involved in athlete’s foot infections [2,14,15]. We sought to increase the antifungal activity of *B*. *subtilis* by engineering it to have increased production of iturin A. To accomplish this, we chose overexpression of the regulator DegQ, as this strategy offered the most straightforward genetic engineering approach and has been used in literature to successfully increase iturin A production in species of *Bacillus* [18,19].

While we found evidence of a more-than 200% increase in iturin A production in our engineered strain in its liquid culture supernatant, this increased production was accompanied by strong growth defects manifesting in a much slower doubling time for the engineered strain. Growth defects have previously been observed when using regulators to increase iturin A production in *B*. *amyloliquefaciens* [19].

Despite the engineered strain producing a higher titer of iturin A, it showed significantly reduced efficacy in an *in vitro* antifungal assay, indicating that it would likely not be suitable for clinical applications. This reduced efficacy may be due to slower growth, potentially decreased levels of other antifungals produced by the cells, or a combination of these and other factors. This result highlights how it is essential to consider more than just total production titer when engineering cells for *in situ* drug production, as growth defects resulting from metabolic burden will affect a vehicle’s ability to both effectively produce drug *in situ* and survive in the competitive skin environment.

Metabolic burden is a known limitation in the field of synthetic biology. Some common strategies to reduce metabolic burden, such as optimization of media and bioreactor conditions, are not feasible when the engineered strain is intended for use *in vivo* [34]. Alternative strategies include careful consideration of potential metabolic burden during the initial strain design phase as well as further strain engineering to control cell metabolism [34]. Another study found that engineering a strain of *Lactococcus lactis* with a circuit to detect *Vibrio cholerae* created a metabolic burden that interfered with the strain’s natural ability to protect against *V*. *cholerae* infection via lactic acid production, but treatment with a combination of the wild-type and engineered strains achieved the desired result of protection and detection *in vivo* [35].

Since several *B*. *subtilis* strains already have the gene cluster for iturin A production, one potential future strategy to increase iturin A production might be direct manipulation of regulatory elements in the genome, which has been done successfully to increase iturin A expression in *B*. *amyloliquefaciens* [19]. Also, while we did not test either strain in an *in vivo* infection model, the performance of unmodified *B*. *subtilis* strain 15841 in controlling growth of *T*. *mentagrophytes* in an *in vitro* antifungal assay suggests that an unmodified strain of *B*. *subtilis* may be sufficient for topical antifungal treatment. For example, other studies have also found that unmodified strains of *B*. *subtilis* performed similarly to the antifungal drug ketoconazole in an *in vivo* mouse infection model [9,10]. A future experiment screening naturally antifungal strains of *B*. *subtilis* in a relevant infection model such as *T*. *mentagrophytes* would help determine if a naturally highly antifungal strain of *B*. *subtilis* could be used to treat fungal infections without the need for engineering increased antifungal activity.

## Conclusion

In this study we engineered *B*. *subtilis* strain 15841 for increased production of the antifungal lipopeptide iturin A by overexpression of the pleiotropic regulator DegQ. We saw an over 200% increase in iturin A in liquid culture for the engineered strain, but also slower growth. In an *in vitro* antifungal assay, we found that the engineered strain reduced the growth of the pathogenic fungus *T*. *mentagrophytes* less effectively than the parent strain. Future studies should seek to balance the side effects of engineering overproduction strains when designing strains for drug delivery applications, as growth defects can be particularly problematic for the desired clinical results. For drugs that are produced in relevant bacteria, the use of strains that naturally produce the desired molecule without engineered overproduction should also be considered.

## Supporting information

S1 Table DNA primers. (DOCX)

S2 Table Annotated iturin A gene cluster for strain 15841. (DOCX)

S1 Raw image Full gel image for amplification of iturin A gene cluster from *B*. *subtilis* strain 15841 genomic DNA by PCR. D/A encompasses a region including ituD and ituA. B1 encompasses part of ituB. B/C encompasses the remaining section of ituB and ituC. Duplicate lanes show replicates of the same PCR reaction. This image was used to generate Fig 1 in the manuscript. “X” marks lanes that were cropped out of the figure. Numbers at the bottom of the image indicate the order in which samples were loaded into gel. The gel was imaged using a UVP UVsolo touch stand-alone gel documentation system.
(PDF)
