# Supplementary material for: Bacillus subtilis engineered for topical delivery of an antifungal agent
Source: PLoS One. 2023 Nov 30;18(11):e0293664. doi: 10.1371/journal.pone.0293664 (PMC10688720; doi:10.1371/journal.pone.0293664)
Supplement: S1 Table — (DOCX) [file pone.0293664.s001.docx]

**S1 Table.** **DNA Primers**

| Name | Sequence (5’-3’) |
| --- | --- |
| ItuDA 5p.F | atgaacaatcttgccttttt |
| ItuDA 3p.R | ctaaaagttcaattgaatagaatc |
| ItuB1 5p.F | atgtcggtatttagaaatcaagaaacgtactgg |
| ItuB1 3p.R | ttccgtcagaaataatgtgatgcatatcaaacatcagaat |
| ItuBC 5p.F | attctgatgtttgatatgcatcacattatttctgacggaa |
| ItuBC 3p.R | ttacattttgtcaaactttgtgtctcccgcaaac |
| pDegQ_RB 5p.F | atgcaatgaaaatttcgtgagcatgcctgcaggtcga |
| pDegQ_RB 3p.R | tcttcaagtttcttttccatgtttgtacacctcctttaagcttgc |
| pDegQ_DegQ 5p.F | cttaaaggaggtgtacaaacatggaaaagaaacttgaaga |
| pDegQ_DegQ 3p.R | gagtcgacctgcaggcatgctcacgaaattttcattgcat |
