# Supplementary material for: Bacillus subtilis engineered for topical delivery of an antifungal agent
Source: PLoS One. 2023 Nov 30;18(11):e0293664. doi: 10.1371/journal.pone.0293664 (PMC10688720; doi:10.1371/journal.pone.0293664)
Supplement: S2 Table — (DOCX) [file pone.0293664.s002.docx]

**S2 Table. Annotated iturin A gene cluster for strain 15841**

| Iturin A | Iturin A gene cluster for *B. subtilis* strain 15841 |
| --- | --- |
| ituD ituA ituB ituC  AAGCTTGTAGGGGCAAAAAGAGCCATTCTCGCCATCGTCTTAATGGGTGCGATTTTAACGTACAGCGGCGTCAGTCTCTTCGTAGCGGTGTTTGCCATTTATCCGTTTGCTAAAAACCTATTTATTAAAGCTGATATTCCCAAGCGTCTGATTCCCGGTACGATTGCATTGGGCGCCTTTACATTTACAATGGATGCGCTTCCCGGCACACCGCAAATTCAAAATGTCATTCCGACCGCTTTTTTTAAAACAAACATTTATGCCGCTCCCTGGCTCGGACTGATTGGTGCAGCATTTGTGCTTACGGCCGGAATGATTTATTTAGAATCCCGGCGCAAAAAAGCGGCCAATGCCGGAGAAGGCTATGCTGGATTTCAATCTGAAGTTTCCGCTGCAGAGGAATCCTCAGGTCTGGCTAATGAAACACATACGCCTGTTTTACCGCAACCCAGCCTCGCGCGGCAAGTGCTCGCCTTTGTTCCGCTTATCCTTGTGGGGGTCATGAACAAATGCTTCACGATGTGGCTCCCGCATTGGTACCCTGACGGATTTGATTTTGCGCCGCTCGGCTTAGAAGCGTTCGGGAAGATTGACCTTTCCTCCGTCACTGCGATCTGGTCAGTAGAACTCGCACTGTTCATCGGAATCATGACAACGATTTTGTTCGATTGGAAAAAAGTCCTCTTCAATTTGAAAGAAGGACTGAATGAGGGCATCGGGGGCGCCTTGCTGGCGTCGATGAATACAGGAGCCGAATACGGATTTGGCGGCATCATTGCGGCGCTTCCCGGCTTTAAAATGGTCAGCAGCGGCATATCGCAGACGTTTACTGATCCGCTCGTCAACGGAGCGGTGACGACCACTGCGCTTGCCGGAATTACAGGATCGGCATCAGGAGGAATGGGCATCGCTTTAAGCACGATGTCAGCAGCGTATACAGAAGCAATCAATACATTCCACATCCCGCCGGAGGTCATGCACCGGGTGATATCGATGGCCTCCGGCGGGATGGACACTCTTCCGCATAATGGCGCCGTCATTACGCTGCTCGCGGTCACCGGGTTGACTCACCGCCAATCCTACAGGGATATCTTTGCCATTACCGTCATTAAAACAGCCGCTGTGTTTGTCATCATCGCCATTTACAGTCTGACGGGCCTTGTGTAGCCAAAACAGAAAGGGGAAACGAATATGGGAAAAGGAAAAGTGATGGACTCTTATCAAGATGCTGCTGCACTGATCAAGGACGGAGACACCCTCATCGCAGGAGGCTTCGGCCTGTGCGGCATTCCGGAACAATTAATTCTCGCGATCAGGGACAGCGGCGTAAAAAACCTGACTGTCGTCAGTAATAACTGCGGCGTTGACGACTGGGGGCTGGGCCTCTTGCTTGCGAACCGTCAGATTAAAAAGATGATCGCTTCCTATGTCGGAGAAAACAAAACTTTTGAACGGCAATTTTTAAACGGAGATCTGGAAGTTGAGCTGGTTCCCCAAGGTACGCTCGCCGAACGGATTCGCGCGGGAGGAGCGGGTATCCCGGCGTTTTACACACCTGCGGGCGTCGGAACATCAGTAGCCGAGGGAAAAGAACATAAGACATTTGACGGACGCACCTATCTTCTGGAGAAAGGGGTTACAGGGAACGTCGCCATTGTAAAAGCGTGGAAAGCTGATCCGCTCGGCAATTTGATTTTCAGAAAAACGGCAAGAAATTTTAATCCGCTCGCCGCTATGGCAGGCAGGGTAACCATTGCCGAGGCAGAGGAAATCGTCGAGGCAGGTGAGCTTGACCCGGATCACATTCATACGCCGGGTATTTTTGTCCAGCATGTGCTGCTCGGCGGGATTCATGAAAAAAGGATCGAACGCCGCACCGTCCGGGAAGCATGATAAAGGAGGCTGATCAAATGAAAGATGGCAGAAAACGAATGATCGCACGGGCAGTAAAAGAAATTAAGGACGGAATGAATGTGAATTTGGGAATCGGCATGCCGACGCTTGTCGCAAATGAAATACCGGAAGATTATAACGTGCTGCTTCAGTCGGAGAACGGCTTGCTCGGAATCGGCCCTTATCCTCCGGCGGGAGCGGAGGACCCGGATCTCATTAACGCCGGCAAAGAAATCATCACCGAAGTTAAAGGAGCCTCTTATTTTGACAGTGCCGAATCGTTTGCTATGATTCGCGGGGGCCATATAGATGTCGCCATCCTCGGCGGCATGGAAGTATCCGAAAAAGGGGACTTGGCCAACTGGATGATACCCGGAAAAATGATCAAAGGAATGGGCGGGGCTATGGACTTGGTTCACGGAGCAAAACGGATCGTTGTCATTATGGAGCACGTAAATAAATTCGGTGAATCTAAGGTGAAATCGGAATGCACTCTGCCTCTGACCGGCAGACAAGTCGTCCATCGGCTGATAACAGATTTGGCGGTGTTCGAATTTCACCAAGGCCGCATGATGCTGACAGAATTGCAGGAAGGTGCGTCATTAGAGGAAGTCATTGAGAAAACAGAAGGTCATTTTACTGTCAGCCAGTCGTTAAAAAACGGGATCAGACCAAAATGAGGAGGTGCCGTCATGCATTCATTGCTTATTGAAAAAACAGCCGTTGTAACAGGTGCAGCAGGAGGAATCGGGCTCGAAATCGCAAAAGAATTCACCCGTGAAGGAGCCGCCGTAATCATCTCTGATGTAAACGAACAAGCGGGGAAAGAAGCGGCGGCAAAACTGACAGATGAAGGCTGTGAAGCAGTCAGCATCACATGCGATGTAACAAATGAAAAGCAAGTGGCCGACATGCTCCAGACGGTCGAGAAACAATTCGGACGTCTCGATATTCTTGTGAATAACGCGGGCATTCAGCATATCGCCCCCATTGAGGCATTTCCCGCGGAACAATTTGAACGGCTCATCCGTGTCATGCTGACCGCGCCATTTATCGCCATGAAACATGCATTTCCCGTCATGAAAAAACAGCAATTCGGACGAATCATAAATATGGCGTCTGTCAACGGGCTCATCGGTTTTTACGGAAAAGCCGCATATAACAGCGCCAAGCATGGCGTCATCGGCTTAACGAAAGTCGGCGCACTTGAAGGCGCGGCTGACGGCATCACCGTCAATGCCCTCTGTCCTGGGTATGTTGACACACAGCTCGTCCACAATCAGCTGAAGGACATTTCTGACACAAGAAATGTGCCGTATGAACGTGTTCTGGAGGACGTCATCTTTCCGCTCGTTCCCCAAAAACGGCTGCTCTCAGTGAAGGAAATCGCTGATTACGCCGTGTTTTTGGCCAGCGACAAAGCAAAAGGCGTCACCGGTCAGGCCGTCGTCATGGACGGAGGATATACCGCTCAATAGTTCTTATACTTTTTGAAAAAACAGCCGCACTGAACGGCTGTTTTTTTTATGCGAAAACCTTGCTCACTCAATAGGCCGATTTACTGCGGCAATCGGTATCCGGGCAGGGTCATTCGCATGTTCTGCGGCGTATTTCGTCCTTGCGGATTTTCCCCATCCAGATCCATCTCTTGCGCCACATTTATAATCAAGCGCAATCAGTTTACAATCCTTCACAGATATACAAATGAATGTTTACAATAAACATAAAATATGTAATTTCTGACACAATTATGCCAATAGACCTAATACATATGAAACATGAAGAGCGCGTCCTAAGTGAAGAAGAGCCGGTAACAGGCTCCACCCCGGCTTTCATCCTCTCTCTTTAAGACTGATTTTCGGTGAAACCCACATGTTTTATTTTATGAATAAATATATCTAATTCGTTTGTAAAATCGAACAATAACTCCTCCGAAAGTATTCTATACACCTTTGTTATAATCATGCTAGGATGTTAGATAAAGGGATATATAGTATGATATTGGATCCCCTGTTCGATGTGATCGGAGGGAACTCATGAACAATCTTGCCTTTTTATTTCCTGGACAAGGGTCTCAATTTGTAGGAATGGGCAAACATTTTTGGAATGAATTTGCGCTTGCAAAGAGATTATTTGAAGAAGCGAGCGATGCGATCTCCTTGGATATAAAAAAGCTGTGCTTTAACGGTGATATGAATGAATTGACAAAGACAATGAATGCGCAGCCCGCTATTTTAACAGTCAGTGTGATTGCTTTTCAAGTGTATATGCAGGAAATAGGGGTTAAACCGCGCTTTCTTGCAGGTCATAGCTTAGGAGAATACTCAGCGCTCGTCTGTAGCGGGGCCCTTTCTTTTCATGATGCCGTTACACTTGTAAGGCGGCGGGGTATTCTTATGCAAAATGCGGATCCTCAGCAGCAGGGGACAATGGCCGCCGTGACGCATCTCTCTCTCCAAACATTACAAGAAATATGTTCGAAAGTGTCGACGGAAGACTTTCCGGCAGGTGTAGCCTGTATGAATTCAGATCAGCAGCATGTGATTTCCGGACACCGGCAAGCTGTGGAACGTGTCATCAGGATGGCTGAGGAAAAGGGAGCTGAATACACTTATTTGAATGTCAGTGCGCCTTTTCACAGTCTGATGATACGATCGGCATCCGAACAATTCCAGACCGTACTACACCAGTATTCCTTTCGGGATGCCGCATGGCCGATTATTTCGAATGTCACCGCACGGCCTTACAGCACCGGCAATTCGGTCAACGAACATCTCAAGCAGCACATGACGATGCCGGTAAGATGGGCGGAATCAATGCATTACTTGCTTTTACACGGAGTCACGGAAGTTATCGAAATGGGCCCGAAGAATGTTTTAGGCAGTCTGCTGAAAAAAATAACGAATCATATTGCTCCTTACCCTTTAGGACAGACATCTGATGTTCACCGGCTGTCGAATTCAGGGGAAAGAAATAAAAATATTGTCCATTTACGCAAAAAACAACTGAATAAATTGATGATTCAATCCGTCATTTCGCGAAATTACAACAAAGATTCATTGGCTTATTCCAACATGACGACGCGATTATTTACGCAAATCCAAGAGCTGAAAAAGAGAATGGAAAGAAATGAAGATGAGCTTTCAGAACAAGAGCTCGAACATTCAATCCATTTGTGCAAATTAATTTGCGAGGCTAAACAGCTTCCGGCTCAGGAACAATTACGGATTTTAAAATAAAGCGCCCAGGAGGGGACCTATGTATACCAGTCAATTCCAAACCTTAGTCGATGTCATTCGGGAAAGAAGCAATATTTCTGATCGCGGAATCCGTTTTATCGAATCCGATAAAAACGAGACAGTTGTCTCTTATCGCCAATTGTTTGAAGAGGCGCAAGGGTTTCTTGGCTATTTACAGCATATCGGCATTCAGCCGAAGCAGGAAATTGTATTTCAAATCCAGGAAAACAAATCATTTGTCGTTGCTTTTTGGGCTTGTTTATTAGGAGGAATGATCCCGGTTCCGGTCAGTATCGGAGAAGATGATGACCATAAGCTGAAGGTCTGGCGGATTTGGAATATATTAAATCATCCGTTTCTGATTGCCTCTGAAAAAGTATTGGACAAAATAAAGAAATACGCTGCAGAACACGATTTACAGGATTTCCATCATCAATTAAACGAGAAATCTGACATTATTCAAGATCAAACCTACGATTACCCCGCTTCGTTTTATGAACCTGATGCGGATGAACTGGCTTTTATCCAATTTTCTTCAGGATCGACAGGAGATCCAAAAGGAGTCATGTTAACGCATCATAACTTAATACATAATACGTGCGCCATTCGAAATGCTCTGTCCATTGACTCAAAAGACTCTTTCTTATCTTGGATGCCCTTAACGCATGATATGGGGCTCATCGCCTGCCACCTAGTTCCCTTCATAGTCGGAATCAATCAAAATCTAATGCCTACAGAATTATTTATTCGCAGACCTATTCTTTGGATGAAAAAAGCTCATGAACATAAAGCCAGTATTCTATCCTCTCCTAATTTCGGATACAACTACTTCCTTAAATTTTTGAAAAACGAACCAGACTGGGATTTATCCCACATCAGAGTCATCGCAAACGGTGCAGAACCGATATTGCCGGAGCTCTGTGACGAATTTTTAACAAGATGCGCAGCATTCAATATGAAAAGATCGGCCATTTTGAATGTTTACGGTTTAGCGGAAGCTTCGGTCGGCGCAGCGTTCTCTAAAACAGGAGAAGAATTCGTTCCCGTTTATCTGCATCGCGACTATTTAAACCTCGGTGAAAGAGCTGTAAAAGTCAGCAAAGAGGATCAAAATTGCGCTTCATTCGTCGAAGTGGGACAGCCTATTGACTATTGTCAGATTCGAATCTCCGACGGAGAAAATGAAAGAATAGAAGACGGAATCATCGGCCATATCCAGATCAAAGGAGACAACGTGACTCAAGGGTATTATAACAACCCCGAAAGTACGGACAGAGCGCTGACTTCTGACGGCTGGATAAAAACGGGAGACCTCGGATTCATTAGTGAAAGTGGCAACTTAGTCGTAACCGGAAGAGAAAAGGACATTATTTTCGTGAACGGAAAAAATATCTACCCTCACGATATTGAACGGGTTGCGATTGAAATGGAAGAGGTTGACTTAGGAAGGGTTGCCGCCTGCGGTGTATATGATCAAAATACGCGAAGCGGAGAAATCGTGCTCTTTGTTGTTTTCAAAAAATCACCTGAAAAATTTGCGCCGCTTGTCAAAGAGATTAAAAAGCATTTGTACCAGCGGGGCGGCTGGAGCATAAAAGAAGTCCTTCCGATCCGCAAGCTTCCAAAAACAACCAGCGGAAAAGTGAAGCGATATGAACTGGCCAGACAGTACGAAGCAGGGGAATTCTCGGTAGAGTCTGCCGCCATCAATGAATTTTTAGAGGGCAGCCCGGAAAAATCAGGACAGACTCCGATTCATGAGATAGAAACGGAATTGCTGTCTATCTTTTCCGAAGTACTGGCCGGGAAAAAGGTTCACCTTACTGATAGTTATTTTGATATGGGCGCAAATTCTTTACAGTTATCGCAGATTGCCGAGCGCATAGAACAGAAATTCGGACGCGAGCTTGCCGTTTCCGACCTCTTTACGTATCCTTCTATCACTGATTTAGCGGCATATCTGTCTGAAGGCCGTGCTGAAATCAAGCCTGATGTGACAGCGGAGCCAAACCATGTTTCTCCGAAAGATATCGCCATTATCGGGATGTCGCTCAATGTTCCGGGAGCATCGACTAAAAGTGATTTTTGGAATCTCCTTGAAAAAGGGGAGCACAGCATTCGAGAATACCCTGTATCTCGGATGAAAGATGCGGCGGATTATGTAAAGGCCATCCAAAGTGATTTCAATGAGAATCAGTTTGTGAAGGGCGGCTATTTAGATGAAATCGACCGATTTGATTATTCGTTCTTCGGGTTAGCTCCTAAAACGGCTCAGTTTATGGACCCTAATCAAAGACTGTTTTTACAGTCTGCATGGCATGCGATTGAAGATGCGGGCTATGCCGGCGACAGTATGAACGGGAGTCAGGTCGGGGTATATGCAGGGTATTCGAAGGTGGGCTACGATTATGAACGCCTCCTTTCTGCGAGTTACCCGGAGGAACTTCATCAATATATTGTAGGCAATCTTCCTTCCGTGTTAGCCAGCCGAATCGCTTATTTCTTAAATTTAAAAGGGCCGGCGGTCACAGTGGATACGGCCTGCTCTTCATCGCTTGCCGCCGTTCATATGGCATGCAAATCTATCATTTCCGGCGAATGTGAAATGGCTATTGCCGGAGGTGTCCGAACATCGCTCCTGCCGATCTGTATCGGGCTTGATATGGAATCTTCGGACGGGTACACCAAAACGTTCAGCAAAGATTCAGACGGCACGGGCACAGGAGAAGGGGCGGCTGCAGTCCTGTTAAAACCTTTGCAGGATGCGGTTCGCGACGGAGACCATATTTACGGTGTGATCAAGGGAAGCGCGATGAATCAAGACGGAACAACTGCCGGCATTACCGCCCCGAATCCGGCAGCTCAGACTGAGGTCATTGAGACGGCTTGGAAAGACGCGGGTGTTGCTCCTGAAACGCTGTCTTTCATCGAAGCGCACGGCACCGGGACCAAACTCGGCGATCCGGTTGAATTTAACGGACTTTGTAAAGCGTTTGAAAAGCATACGTCAAAAAAACAATTTTGTGCGATTGGTTCTGTTAAATCAAACATCGGTCATTTGTTTGAAGCGGCAGGCATCGTGGGGCTGATCAAATCTGTCCTCATGCTGAATCATAAGAAAAACCCGCCGTTAGCGCACTTTAATGAACCTAATCCGCTCATTCACTTTCACTCTTCACCTTTTTATGTAAATCAGGAAGCTGAGGAGTTCACACCTGGTGACGGGCCTCTGCGGGGCGGAATCAGCTCGTTTGGTTTTAGCGGAACGAATGCGCATGTGGTGTTGGAGGAATATACTGCCCAAAATGAGTATGCTCCCGAGGACGAAAATGAGCCGCACCTATTTGTTTTGTCTGCTCAAACAGAAAATTCACTCTATGAGCTCGTACAACAGTACCGGCAATATGTATCGAATGACAGCCAAGCCTCATTGAAGTCCATTTGCTATACAGCCAGTACGGGCAGGGCTCATTTGGATCATAGGATTGCCATGATTGTATCAAGTAAACAAGAGTTATCTGATAAGCTGACCTGTCTGGTTCAGGGAGACAGAAATATCCCCGGAGCATACGTTGGTTATCAGAATATGAAGGAAATGCTGCCTGCTCATAAAGAAGAGCTGAATAAACAAACCGCTGATCTGATCAAGCGGAGTACACATACACAGGATGAACGAAACACACGGCTGAATCGCGCCGCCGAATTATTTGTGAAAGGAGCCGTTATAGATTGGCGCGCGTTTTATTCCGGGGAAACCGTACAAAAAACGCCATTGCCTTTGTATCCGTTTGAACGGAATCGCTGTTGGGCTGAAGCTGCCCCTTTGAGCGTAAACGAGGGAGAAGAGAGAGGAGAAGCAGTATTGAATATCAATCAATCGAAGGCGCATATTGAATCCTTCCTGAAAACTGTAATCAGTAATGCTTCGGGGATCAGAGCGGATGAAATCGATCTGAATGCCCATTTTATCGGACTCGGAATGGATTCCATCATGCTGTCACAGGTCAAAAAGGCCATTGCGGACGAATTTGGGGAAGACATCCCGATGGATCGTTTTTTTGATACGATGAACAACCTTCAAAGTGTCATAGATTACTTGGCTGAGACCGCTCCAACATCCTTTGCATCCGCTCCGCCTCAAGAAAATGTTCCGGCGCGAGAAAAACCGGTCATTTCAGAAGCACAGCCTGAGTCGGAGCACAGAGAAGACCGCCAAGAGCATATGCTCGAAAAAATAATCGCTTCTCAGAATCAATTAATTCAGGATACCTTACAGGCTCAATTAAATAGCTTTAATTTACTGAGAACCAGCGGGCATCATTCCGATGTGAAAGAATACTCTAAAGCGCAAAAAAAATCAATTTCTTCAGTCGAGCAGGGGCCTCCTGCTGTCACTGCAGAAAAGAAATTGACTCAAGAAGCGAAACCCTATGTTCCTTTTCAGCCTCAGAGCCTGCATGAACAGGGACACTATACCGCACAGCAAAGACAATATTTAGAAGATTTCATCAGGAAATACGCAGACAAAACGAAAGGTTCCAAACAATATACGGACAAAACACGATTTGCTCATGCAAACAACCGTAATCTGTCCAGCTTCCGTTCATATTGGAAGGAAATCGTGTACCCGATTATTGCCGAACGTTCTGACGGTTCCAAGATGTGGGATATTGACGGAAACGAATATATCGATATCACCATGGGATTCGGAGTTAATCTTTTCGGACATCATCCATCGTTTATTACACAGGTGATCGATGATTCAACCCGCTCATCCTTGCCACCGCTCGGACCGATGTCGGATGTCGCCGGGGAAGTTGCCGACCGAATCCGCACATGCACTGGCGTAGAAAGGGTCGCTTTCTATAATTCCGGAACAGAGGCCGTCATGGTTGCCCTACGTTTGGCAAGGGCGGCAACAGGAAGAAAGAAAGTTGTGGCGTTCTCGGGCTCTTATCACGGGACGTTTGACGGCGTATTGGGAGTTGCGAGCACAAAAGGCGGGGCTGCGTCTGCGAATCCGCTGGCTCCGGGCATACTGCAGAGCTTTATGGATGATTTGATTATTTTGCATTACAACAATCCAGATTCGCTGGACGTGATTCGCAGTCTCGGGGATGAATTGGCCGCCGTACTGGTGGAACCGGTACAAAGCCGCAGACCGGATTTGCAGCCGAAAGCATTTCTGAAAGAACTGCGGGCGATTACACAGCAATCCGGGACGGCTCTGATTATGGATGAAATTATTACGGGATTTCGGATCGGTCTCGGCGGCGCGCAGGAATGGTTTGGCATTCAAGCCGATTTAGTGACCTACGGAAAAATCATCGGAGGCGGACAGCCGTTAGGGATTGTTGCCGGAAAAGCTGAGTTCATGAATGCGATCGATGGGGGAACCTGGCAGTATGGGGATGATTCCTATCCGCAAGACGAGGCGAAACGCACCTTTGTGGCCGGCACCTTCAATACTCACCCGCTTACCATGAGAATGTCATTAGCCGTGCTTCGCCATTTACAAACCGAGGGAGAACACCTGTATGAGCAGTTAAATCAAAAAACAGCCTATCTGGTCGATCAGCTAAATCGCTGCTTTGAACAATCAGAAGTGCCTATCCGCATGGTTCGATTCGGTTCTTTATTCCGGTTTGTTTCATCGCTTGATAACGACTTGTTCTTTTACCACCTCAATCCTAAAGGGATCTATGTATGGGAAGGACGCAACTGCTTCTTGTCCACGGCGCATACCGCGGATGATATTGAAAACATCATTCAAGCCGTTAAAGACACGGTGAAGGATCTTCGCCGAGGCGGATTTATTCCGGGTGGCCCGGATTCCCCTGATGACGGAGGCCATAACAAAACCGGAATACACGAGCTTTCACCTGAACAAAAACAATTGGTTCTGGCATCACATTATGGGAATGAAGCTTCTGCAGCCTTAAACCAGTCCATTATGCTGAAAGTGAAGGGGAAACTGCAGCATACGCCCTTAAAACAAGCAGTCCGGCATATCGTCGGCCGCCACGAGGCTTTACGTACGGTGATCCATGTTGATGACGAAGTACAGCAAGTGCAGGAGCGGATAAATGTAGAAATTCCTGTCATTGATTTTACCGGTTACCTGGAAGAGCAACGGGAGCCGGAAATTCAGAAATGGCTGCAAGAAGATGCCAAGCGGCCGTTTCATTTCCAAGAACAAAAACCCTTGTTTAGAATCCATGTGCTTACATCGGGCCAAGACGAACATCTGATTGTGCTTACATTCCATCATATCATTGCCGACGGATGGTCAATTGCTGTCTTTGTTCAAGAGCTGGAGAGCAGCTATGCGGCAATGGCAGAAGGAAAACCGATTCCTCCGAAAGCGGCCGCTGCTTCATTTCGCCAATATTTAGACTGGCAGCAGGCCCAGATTGACAGCGGCCATTATGATGAAGGAGTCCGTTATTGGCGTCACCATTTCTCTGAACCGATACAGCAGGCAGTCCTGCCGAGCATCGCTTCTGCCCGTTTTCCGAACGGGTATGAGGGTGACCGGTTCACCGTCAGGCTTGGGCGGCCTTTAAGCGAGGCTTTAAAGTCATTAAGCATTCGGAGGAAAAATAGCGTATTTGTGACGATGCTGGGGGCATTTCATCTTTTTCTGCACCAGCTTACCAAACAGTCCGGTCTTGTGATCGAAATTCCTACAGCCGGTCAATCGCATATGAAACAGCATGATCTGATTGGAAATTGCGTCAATATGATTCCGGTAAAGACCACATCTTCTTCAGAAAGCACCTTATCCGGTTATCTTGGCAGTATGAAAGAAAGCGTGAACCATGCAATGCTTCATCAGGCCGTTCCAATGACACTGATAGCCAGAGAACTTCCGCACGATCAAGTGCCGGAAATGCGTATTATTTTTAATTTGGACAGGCCTTTTCGAAAGCTTCATTTCGGAAAGGCGGAAGCCGAGCTCGTTGCTTACCCTATGAAATGCATTTCATATGATCTATTTCTTAATGCTACGGACGCACATCAAGAATATGTTCTTGATTTCGACTTTAATACGAATGTCATCAGTCCGGAGATCATGAAAAAGTGGGGAGCAGGCTTTACGAAATTACTGCAAAAAATGGTTGAGGGGGATTCAGTCCCTCTTGACGCCCTGACGATGTTTTCCGATGAAGAACAGCATGATTTACAACAACTGTATGCCGACCATCAAAATCGGATCTCTTCAATAGTCAGCAATACGGCAAACTTCGCTGACGCCTACGAGGCGCCGGCAAATGAAACGGAGCGGCAGCTGGCGCAGATTTGGGAGGAACTTTTCGGCCTTGACCGGGTTGGCAGATCAGATCGCTTCCTGGCATTGGGAGGAAACTCACTCCAAGCGACGCTTATGCTTTCCAAAGTTCAGAAGACATTTCATCAAAAGGTTTCCATCGGACAATTTTTCAATCACCAGACTGTTAAGGAATTAGCAAGTTTCATTCAGAATGAAAAGAAAGTCATGCACCTCCCGATGAAGGCGGCTGAGAAAAAAGCGCATTACCCGACATCACCGGCGCAGCAAAGAGTATATTTCCTTCACCAAATGGAACCGGATCAGCTGGCTCAGAATATGTTCGGCCAAATATCAATTATAGGGCAATACGATGAGCAAGCCCTGATTTCTTCTCTTCAGCAAGTCATGCAGCGGCATGAAGCGTTTCGCACGTATTTTGACATTTCAGATGGCGAAATTGTCCAGAAACTTGAAAACGAAATTGATATTAACATTCATGTCCGGACAATGAGTCTGGACGAATTTGATGCCTATGCAGACCGGTTTGTAAAACCGTTCCGCTTGGAGCAAGCTCCTTTGGTCCGTGCGGAGCTGATCAAAATTGATAACGGGCAGGCCGAGCTGCTCATCGATATGCACCATATCATTTCGGACGGTTATTCCATCAACATACTTACAAATGAATTGCTGGCTTTATATCATCAGAAACCATTACCGGACATTGAATTTGAATATAAAGATTTCGCAGAATGGCAGAACCAATGGCTGAATGAGGATGCCATGAAGCAGCAGGAGACATACTGGCTTGAACAATTCCAAGAAGAAATCCCGGTTCTTCAACTGCCGACAGACGGTTCAAAAGCGGCGGAACGGTCTTCCGACGGGCAGCGCGTAACGTGCTCCTTACAGCCGGATGTAATCCGTTCGCTGCAAGATCTGGCGCAAAAGGCGGGAACGACGCTTTATACGGTGCTTCTGGCCGCCTACAAAGTGCTGCTTCATAAATATACAGGACAAGAAGATATTGTCGTAGGCACGCCTGCTTCAGGAAGAAATCATCCGGATGTCGAAAATATCATCGGGATTTTCATTCAAACCATCGGAATCCGGACGAAGCCGCTCGCTAATAGAAAGTTTACGGATTATCTGGAAGAAGTAAAGCGGCAGACGCTTGACGCTTTCGAAAACCAAGACTATCCATTCGACCGGCTGGTGGAAAAATTAAATGTACAACGGGAAACAACAGGCAAATCACTGTTTAACACAATGTTTGTGTTTCAAAATATTGAATTTCATGAAATCCGGCACGATGAATGTACATTTAAGGTGAAAGAACGAAATCCAGGGGTCTCTTTATATGATTTGATGCTGACGGTTGAAGATGCCGGGCAACAGATTGAGATGCACTTTGATTTTAAACCGGGACAGTTTGAAAAAGACACCATTGAACAGATCACAAGACACTATGTCAGTATTTTAAAGAGCCTTGTGAAGCAGCCGGAGATGACGTTGTCTTCCGTTCCTATGCTGTCTGAAACCGAACGGTATCAATTGCTGACGGAGTTTAATGACACAAAGACTCCGTATCCGCATAACGAAACGGTATCCCGGTGGTTTGAAAACCAGGCAAACCTGCGGCCTGATCATGAAGCCGTTATTTTCGGCAATGAGCGGTATACGTACAGACAGCTCAATGAACGGGCAAACCAATTGGCGCGGACGTTACGGACAATAGGCATACAAGCGGATCAATTCGTTGCCATCATCTCTCCGCATTGTATCGAGCTGATTGTTGGAATTTTGGCTGTTCTGAAATCCGGCTGCGCATACGTGCCCATTGATCCTGAATATCCGGAGGACCGGATCCATTATATGCTGAGTGACTCAAAAGCGGAGATTGTGTTGACACACCGCAGCCTGCAAGTTCAGTTACCGTATGATGGTGACGTTGTGCTTCTGGATGAGGAAAACTCTTATCATGCAGACCGCTCAAATCTTGAATTGTTCAGCGATGCGCATGATTTAGCGTACATGATCTATACGTCGGGTTCGACCGGCAATCCGAAAGGTGTACTCATTGAGCACCAGGGGATGGCCAATTATATTTGGTGGGCGAAAGAAGTTTATGTGCGGGGAGAGAAAACCAACTTCCCATTATACTCTTCCATCTCTTTTGACCTGACGGTGACTTCGATATTTACACCGCTGGTGACGGGAAATACCATCATCGTCTATGACGGAGAAGATAAAAGCGCTGTTCTTTCTGCGATTATGCAAGACTCAAGAATAGACATGATCAAATTGACCCCGGCACATTTGCACGTCATCAAGGAGATGAATATAGCAAGTCACACCACAATACGGAAAATGATTGTAGGCGGAGAAAATTTTAGCACCCGTCTGGCCAAAAGTGTCAGCAAGCAGTTTAAAGGACGGCTGGACATATTCAATGAGTACGGTCCGACGGAAACTGTCGTCGGATGTATGATTTACCGTTACGACGCAAAACGGGACAAGCAGGAATTTGTACCGATCGGCACTGCGGCTGCCAACACGGACATTTATGTGGCTGATGCAAGCAGAAATCTGGTTCCAATCGGGGTAATCGGTGAAATGTATATCAGCGGACCCGGTGTTGCCAGAGGGTATTGGAATCGCCCGGATTTAACGGCAGAAAAATTTGTTGAAAACCCGTTTATCCCGGGAGCGAAGATGTACAAATCAGGGGACTTGGCTAAGCGATTGAGGGATGGAAATCTTGTATATGTGGGCCGCATCGATGAACAAGTCAAAATCAGGGGACACCGAATCGAACTTGGCGAGATCGAAGCAGCGATGCATAACGTGGAAACGGTGCAAAAAGCCGCGGTTACAGCCAGAGAAGCAGAAGACGGCTTAAAACAATTGTGCGCGTATTACGTAAGCGACAAACCTATAGCGGCTGCGCAGCTTAGGGAACAATTGTCATCGGAGCTCCCGGACTATATGGTTCCGTCCTATTTTGTCCGTCTTGAGCATATGCCGTTAACGTCCAACGGGAAAATAAACCGTAAAGCACTGCCTGCACCCGAAGCAAGTCTGCAGCAGACAGCTGAATATGTTCCGCCGGGCAATGAGGTCGAGTCCAAACTGACAGATTTGTGGCAGGAAGTGCTCGGAATAAGCCGTGCGGGGATCAAACATAATTTCTTTGATCTCGGAGGAAATTCCATTCGTGCAGCGGCCTTAGCCGCACGAATTCATAAAGAGTTGGATGTGAACCTGACGTTAAAAGACATATTCAAGTTTCCTACCATTGAACAATTGGCTGACAAAGCGTTACACATGGGCAAAAATCAATATGTACCGATCCCGGCCGCAAAGGAAATGCCCTATTATCCGGTTTCTTCAGCTCAAAGGCGCATGTATTTGTTAAGTCACACAGAAGGCGGCGAGCTGACTTACAATATGACGGGTGCCATGAGTGTGGAAGGGACGATCGATCCCGACCGGTTAAACGCCGCTTTTCAAAAATTAATCGAACGTCATGAAGCTTTGCGGACAAGCTTCGAATTATACAAAGGCGAACCGGCACAGCGTATTCATCAGAGCGTCGAATTTACAATAGAACGAATTCAAGCTAGCGAAGAAGAAGCGAAAGACCGTGTGATTGATTTCATCCAAGCGTTTGATTTAGCCAAGCCGCCGCTGATGCGGGCCGGTCTGATTGAAGTTGAACCTGCGCGGCATGTGCTTGTGGTTGATATGCATCATATCATTTCTGACGGGGTGTCCGAGAATATTCTGATGAAGGATTTAAGCCTGCTTTACGCGGGGAACGAACCGGAGCCGCTTTCTGTTCAATATAAAGACTATGCGGTTTGGCAGCAATCTGACATTCAGAAACGGAATATCGAGAGCCAGGAAGCGTATTGGCTGGATCAGTTTCGTGATGACATTCCTGTACTGGAAATGCCTGCGGATTACGAGAGACCTGATATACGCGATTACGAAGGAGAATCCTTTGAATTTGTTATACCGGAACACTTGAAACAGCGTTTAAGCCAACTTGAAGAAAACACAGGAGCAACACTGTATATGATTTTATTGGCAGCCTATACGATTCTTTTATCCAAATACAGCGGACAAGAGGATATTGTCGTAGGAACGCCATCTGCCGGGCGGACTCATTTGGATGTAGAGTCAGTCGTGGGAATGTTCGTCAATACGTTAGTCATTCGCAATCACCCGGCGGGCCGTAAAACATTTGATGCCTACTTAAACGAAGTAAAGGAAAACATGCTGAATGCCTATAAAAATCAAGACTATCCGTTGGAAGAATTGATTCAGCACTTGCATTTCCCAAAAGATTCAAGCCGCAATCCTTTGTTCGATACGATGTTTGTGCTGCAAAATCTCGATCAGGCTGAATTGACATTCGATTCCCTTCAGCTCAAGCCGTATCAATTTCATCATCCGGTTGCCAAATTCGATCTGACCTTGTCGATTCAGGCGGACCAGGACAACTATCACGGTCTGTTTGAATATTCTAAAAAGCTGTTTAAAAAAAGCAGAATCGAGGCTTTATCAAACGACTACTTACACATCCTATCAGCGATTCTAGAACAACCGAACATAACAATCGAATATATCGGGTTGAGCGGCGGCGATGAGGAAGAAGAGAACATGCTGGATTCTATTCAATTGAACTTTTAGGGAAAAGCATTATTTCCTTAAATTATACAAAACGAGGTGCTCCAATGTCGGTATTTAGAAATCAAGAAACGTACTGGGATAACCTGTTTGATGAAGATGACGGCCTAAGCGTATTCCCTTACTTCAAAGCAGAGGATAATGCGTCATTGGCCCGTATCGGCTATCAGGAAAAATGCATCTGCCGTTCCTTATCTCCGGAAGTATCTCAGAGAATCATGACAATGGCGAATCATTCCGAAATGGCAGCCTATTTGATTTTATTGGCTGGTATTGAATGCTTGCTGTATAAATATACGGATCGAGCGAGTTTGATTCTCGGCATTCCAACGGTATCGAAGCAAAAAAGCAGCCAGTCAGCCGTTAATACCATCGTCCTCTTAAAAAATACGCTTACAAGCGAAAGCACGTTCAAAAGCGTATTCCAGCAACTGAAAGAAGCCGTTAATGATTCGCTGAAAAATCAAAACCTGCCGTTCCGGAAAATGGTTCAGAATCTAAACGTGCAATACAATGATGAGCATATTCCGTTCATTCATACCGTCGTTTCGCTTAACGAAATTCATTCCAAGCAATTTAAGGAAGACGCTGCAACCGATACGCTGTTTCACTTTGATATGGAGAACAACGGGGTTCACTTGAAGCTTTTTTATAACGGCAATCTGTACGATGAGCGCTATATGGATCAAATTGCAGCCCATTTGGATCAGCTGCTGTCCGTGATCTTGTTTCAGCCTCAAACTGCAATCCATACGGCAGAAATGATACCCGAGACGGAAAAACAGAAACTATTGTTTGATTTCAATGACACCGGCGCAGATTACTCCGGGAGCAGAACCGTTTATCAATTATTTGAAGAGCAGGCAGAAAGAACGCCCGAGCATCCAGCCGTGAAGTTTAAAAACTGCCATTTGACGTACAGAGAATTGAATGAAAGAGCCAACCGTTTGGCAAGAACATTGCGCAACTGCGGGGTTCAAGCCGATTCGTTGGTCGCTATTCTGGCTGATCGTTCATTGGAGATGATCGTGTCTATTATAGCCGTTTGGAAAGCGGGGGGCGCTTATGTGCCCCTGGACCCTGAATATCCAAATGAGCGGCTTCAATATTTGCTTCATGATGCGAATGCAGATGTTCTCCTTGTACAGCATCATTTTAAAAACAGCCTCACCTTTGACGGTCCGACGATTGATCTGAATGATGAAACATCTTACCATGCCGATTGCTCATTGTTATCACCTATAGCCGAACACAGCCATTTAGCCTATGTCATCTATACTTCAGGCACAACAGGAAAGCCGAAAGGGGTTATGGTTGAACACGGCGGCATTGCGAACTCGCTGCAGTGGAAGAAGGCGTTTTTTAAGCATTCTGCCAAGGACCGTGTGCTTGTCTTGTACCCCTATGTTTTTGATGCATTTATTTTAAATTTCTTCGGCCCGCTTATTTCGGGGGCAACCTTGCATCTTTTGCCGAATGAAGAGAATAAAGACCTTTTCGCCATTCAAAATGTCATGAAACAGGAGAGAATTACACATTTCTCAACTTCTCCCCGTCTTCTGAAAACGATGATCGAACAGATGAACGCAGAGGACTTCATTGATGTCCAGCATGTTGTTGTAGGCGGAGAGCAGCTGGAAACAGATACAGTTGAGAAGCTGTATTCTCTGCAGCCTCATATTCGAATCAATAATGAATACGGACCGACAGAAAATAGCGTGGTTTCTACGTTTCACCCTGTTCAGTCCACTGATGAACAGATCACAATCGGCAAACCGGTGGCTAACCATCAAGCCTATATTTTAGGAGCGCACCAGCAAATCCAGCCGATCGGCGTACCGGGAGAGCTGTATGTCGGAGGAGCGGGAGTGGCCCGCGGCTACCTCAATCGGCCTGAATTGACGGAGGAAAAGTTTGTTCAGCATTTGCATCTCCCCGCTCAAAAGATGTACAAAACGGGTGATCTCGCAAGATGGCTGCCAGATGGAAGGATCGAATATTTAGGACGAATTGATCACCAAGTGAAAATTCGCGGCTACCGGATTGAGATCGGCGAGGCAGAGGCAGCCATGTTCAATCTGGAGAACGTTCGTGAAGCTGCAGTCGTTGCGCGGGAAGACGAAGACGGAGCCAGGCAATTGTATGCCTATTATGTCGGCGAGCCTTCTTTGACCGCCGCGCAGTTCAGAGACGAATTATCCCGTAAACTGCCGGATTATATGATTCCCTCTTACTTTATCCATTTGGAACACATACCGCTCACTTCCAATGGAAAGATTGATTTGAAAGCTTTGCCTGCTGCCGAAGAAAAAACGCGGACGGAAAATGAATACATAGCCCCGCGAAACACAACAGAAGAACTGCTGGTGTCTATCTGGCAGGAAGTATTGGGGGCAGAACGTATAGGAATTCTGGATAATTTCTTTGATTTTGGCGGAGATTCGATTAAATCAATTCAGGTGTCTTCCAGATTGTATCAAGCCGGATACAAGGTTGATATGAACAATTTGTTTAAATATCCCGCGATTGCTGAACTAAGTCCGTTTGTAGTGCCGGTCAGCAGAATGGCCGATCAAGGGGAAGTGAGCGGCAGGACAAATCTGACTCCGATTCAGCATTGGTTTTTTGAGCAAAAAATGCCGCATGCACACCATTACAATCAGGCCGTCATGTTATATTCGGCGGAAGGCTTTAAAGAGGATCCGCTTCGCCGGACGATGGAAAGCATCGCATCGCATCACGATGCCTTAAGGATGATTTTTGAAGAAACGCCGAATGGATACACCGCGCGAATCACAGGAACGGATGAACATGAATTATACCATCTGGAAGTGTTGAATTATAAAGAGGTAACAGATCCGGCTCAAGCAATTGCCGAAAAAGCGAATGAGATTCAAAGCAGCATGGTATTAGAGCAGGGGCCTTTAATGAAGCTTGGTTTGTTCCAATGTCCGGACGGAGATCATTTGCTGATTGCGATTCACCACTTGGTGATTGACGGGGTATCATGGCGAATTTTACTTGAGGATTTTGCAAGCGGTTATGAGCAGGCAGAGCAAGGGCAAACGATCCGCCTTCCGCAAAAAACAGACTCCTTTCCATTCTGGGCTGATCAATTGTCCAAATATGCGAAGGAAACCGACTTGGAGCAGGAGATTGCCTATTGGTCCGAGCTTTCAAGCACGAAGCCGCAGCCATTGCCTAAGGACAAAATAAGCGAAGGTTCGTTATTAAGAGACAGCGAAGAATTGACGATTCAATGGACAAAAGAGGAAACAGAGCAATTATTAAAACAAGCGAACCGCGCATACAACACGGAAATCAATGATCTGCTGCTGACTTCATTAGGACTCGCCGTTCACAGGTGGACTGGCATGGAAGACATTGTCGTAAATTTGGAAGGCCACGGGAGAGAACCGATTATTCCGGATGCGGATATTTCCCGGACGATAGGATGGTTTACAAGCCAGTACCCTGTCGTACTGAGGATGGAAGCCGGCAAAGACCTGTCACAGCGAATTAAGGCCGTGAAGGAAGGGCTGCGCCGTATCCCTGATAAAGGAATGAATTACAGCATCATTAAATATGTATCCGGGCGTCCGGAAGCGGAAACCCTTCAGCTGAATCCGGAAATATCGTTTAATTACTTAGGGCAATTTGACCAAAATCTGAAACATCAAGCTTTGCGCATATCGCCTTTTTCCACTGGGTTATCGATGAATGAAAACCAAGAGAGACTGGCCGTGCTTGATGTAAGCGGCATGATAGCTGAAGGGAAATTATCGCTGACACTGAGCTACAGCAGCAAACAATACGAAAAATCGACTATGGCGCAATTTGCCCGAAGTTTGAAAGAAAGTCTGCAAGAAGTCATTGCGCATTGCGTAAGCCGGCAGCAGACTTCCCTGACGCCGAGTGACATCCTGTTAAAGAACATCACAATAGATGAACTGGAACAACTTTTGGAGCAGACACGTGAATTGGGCGAAGCGGAAAATATTTATCCGCTAACACCGATGCAGAAGGGTATGCTGTTTCACAGCTTGTTTGACCCGAGTTCAGGCGCTTATTTTCAGCAAACGATGTTTGATCTGCATGGAGATCTGGATATTGATTCATTTTCGAAAAGCTTGGACGGCTTATCGCAAAAATACGATATTTTTCGCACGAACTTTTACAGAGGCTGGAAAGATCAGCCGCTGCAAATCATCTTAAAAACGAAAAAGATCGGTTTTCAATTCATTGACTTACGTGAGATGAAAAAATCACAAAAAGAAGAGATGATTCAGAAATACGCCAGAGAAGATAAGTTACAGGGGTTCGACTTAGAGAAAGGCGAGTTAATGCGGTTACTCATTCTTCGTACGGATGATACGGCATACCGTTTTATATGGAGTTTTCACCATATTTTGATGGACGGCTGGTGCCTGCCGCTGATCACAAAAGAAATATTCGAACATTACTTTGCTTTGCTTCAGCAAAAGCAGCCTGAGCAATCGTCAATTACTCCCTACAGTCGATTCATTGAATGGCTCGGCCGGCAAGATGCAGAAGAGGCCATGCGTTATTGGGATCAGTATTTAGAGGGATATGAAGAGCAAACCGGCCTTCCCAAGGATCACCATTCGGCGGATGACGGGCGATACATTCTCGAGAAAGTGACGTGTGAAATAAGCTCTGATCTCACTTTGAAAATGAAGCAGACGGCCGGCAAGCGCCACGTCACGCTGAATACGCTGCTGCAGACGGCTTGGGCGGTTCTGCTGCAGAAATATAACCGGAGTAAAGACGTTGTTTTTGGAAGTGTCGTTTCCGGAAGACCGGCTGGAATTCCAAACGTGGAAACCATGATCGGCTTATTCATTAACACCATTCCCGTCCGAATCCGATGTGAAGCCGGAACAACGTTTGCCGAGCTTATGAAAGAGGCGCAGGAAAGGGCAGTAGCGTCGCAAAAATTTGAGACGCACCCGTTGTACGATATTCAGGCGCGGACAACGCAAAAGCAAGATTTAATTACACATTTAATGATATTCGAAAATTATCCCGTTGATCAGTATATGGAAAGCATAGGCCGGCAAAACGGATCATCCATCACCATTTCCAACGTGCAAATGGAAGAACAGACGAACTATGATTTTAATTTGACCGTTATACCGGGTGACGCGATGAACATCTATTTTGAATACAACGCCAATGTGTATGATCGATCAAGTATAGAACGGATTCGGGAGCATTTCATGCAAATTTTGCATCAAGTCGTGACAGATGCGGATATCCTGGTGGAGCAAGTGGAATTGTTAACGGAAGGTGAAAAAAGAACCCTTCTTCATACGCTTAACGACACGTCTGCGCCATTTCCGCAAATACCGGTTTATCAATTATTTGAAGAGCAATCACAGCGCACGCCCGATCAAGCGGCCGTGATCGATAAGGACAGGCAGCTCACGTACGGAGAGCTCAATAAGCGGGCAAACCAATTGGCGAGAACGTTAAGAGCGAAGGGCGTGCAGGCGGATCATCCTGTGGCTGTCATCAGCCGAAACAGTATCGAATCGGTGGTCGGAATCCTTGCCGTTTTAAAATCAGGCGGAGCCTACGTCCCTATCGATCCGGAATATCCGCAAGACCGAATTCGGTATATGCTGGATGACTCTCAAGCCGAAATCATTTTGATGCAGCGGGATGTCAGGGAGCAGCTCTCCTGCGAAGGCGTCACGGTTTTACTGGATGATGAAAGTTCGTATCACCAAGATGACGCTGACCTTGAACCGCTAAGTGATGCGAGCCATTTGGCGTATGTCATTTATACGTCAGGTTCCACAGGCCGGCCGAAAGGCGTATTAATCGAGCACCGGGGCTTGGCCAATTATATTTGGTGGGCGAAAGAGGTTTATGTAAAAGGCGAGAAAACCAACTTCCCGTTGTATTCGTCAATCTCTTTTGATCTGACGGTGACTTCTATCTTCACACCGCTAGTCACGGGCAATACGATCATCGTCTACGACGGTGAGGATAAAACAGCACTGATTGCATCAATTGTGCAGGACCCGAGGGTGGATATCATTAAATTGACCCCGGCCCATTTGCAGGTTTTAAAAGAAATGAACCTCGCCGATAAAACGGCTGTCCGAAGAATGATTGTGGGCGGGGAGAATCTAAGCACCCGGTTAGCCCAAAGTATTCATGAACAGTTCGAAGGCCGGATTGAAATATGCAATGAGTACGGGCCGACTGAAACCGTTGTCGGCTGCATGATTTACCGTTACGATGCCGTAAAGGACAGGCGGGAATCGGTACCGATCGGAACCGCTGCAGCAAACATGAGCATTTATGTCCTTGATGAGAATAGGAAACCCGTGCCGGTCGGTGTCCCTGGGGAAATCTATATCAGCGGCGCCGGCGTGGCAAGAGGATATTTAAACCGGCCGGAATTAACGGCAGAGAAATTTGTCGATGACCCGTTTGAACCCGGAGCCAAGATGTATAAGACCGGAGATTTGGCGAAGTGGCTGGCCGACGGAAACATCGAATATACGGGGAGAATAGATGAACAAGTAAAAATCAGAGGCTATCGTATCGAATTAGGAGAAATCGAGGCGGCACTGCACCAAGAAGAAGCAATCAAAGAAGCGGTCGTGACAGCCAGAGAAGATGTCCACGGGTTCAAACAATTGTGTGCATATTACGTGAGCTGCGGACAAATAACGGTATCCAAGCTTCGAAAACAATTGTCTCAAACCCTGGCAAGTTATATGATTCCGGCTTATTTTATCGAGATGGATGAAATGCCTTTAACCTCCAACGGAAAAATAAACAGAAAAGGGCTGCCGGCTCCGGATTTCGGGCTGCAGGACAGAGCAGAGTATAAGGCTCCCCGAACGAAAGCGGAAGAAATATTAGTTTCCGTCTGGGAATCGGTATTAGGCGCAGAGAACGTCGGTATTCTGGATAATTTCTTTGATCTCGGCGGAGATTCGATTAAATCAATTCAAGTGTCATCCAGATTAAAACAGAGCGGATACAAAATGGAAATCAAAGACTTGTTTCAATATGCAACGATTGCCGAATTGAGTCCGCATATCAAGCAGAATGTACGTATAGCCGATCAGGGTGAAGTCAAAGGAAAAGTCAGCCTGACTCCTATTCAGCATTGGTTCTTTGATCACATAACGGTAGATCCGCATTATTATAATCAAGCCGTCATGCTGTTTGCGCCGGAAGGTTTTCAGGAAACGCCGCTTCGTCAAACCCTGCAAAAGCTCGCTGAACATCACGACGCGCTTCGCATGACGTTCCGGCAGACAGAAAAAGGATACGAGGCACAAAATGCAGAGATCGGCCAAAGCAGGCTGTACCACCTTGAAGTCCTGAATCTGAAAGCAGAGTCCGACCCGGGGCAGATAATTGAGGCCAAGGCTGACGAGATACAAGGCAGTATGCGTTTAAGTGACGGGCCGTTAATGAAAGCCGGGCTGTTTCAATGTGCAGATGGAGATCATTTACTGATTGCCATTCATCATTTGATTGTAGACGGGGTTTCATGGCGCATTTTATTGGAGGATATCGTCAGCGGCTACAGGCAAGCTGAGAACGGGCAAGTGGTTCAACTGCCGCAAAAAACAGACTCTTTCCAATTATGGGCCAAGAGACTTTCAGAATATGCGCAAAGTGAAACGATAAAACAAGAACAGGAGTATTGGACAAAGATCGAACAAACCGAAGTAAAACCGCTGCCTACAGATTTTCATGAAACACAGACTACTGCAAAAGACAGTGAAACGGCAGCTGTGGAATGGACTAAAGAGGAAACGGAGCTGTTATTAAAACAAGCGAATCGCGCCTATAACACTGAAATAAACGATTTGCTTTTAACTTCCCTTGGTCTTTCCATATCACACTGGTCAGGACTTGAACACATTCCGATTCATTTAGAGGGACACGGAAGAGAACAGATTTTTCAAGATATGGATATCTCCCGCACGGTTGGATGGTTTACAAGCCTGTATCCGGTTGTGCTTCACGCGCAGCCAGGCAAAGAAATCTCTGATTATATCAAGATGACCAAAGAAGGGCTGCGCCAAATTCCGGATAAAGGGATCGGATATGGGATAGCAAGGTACTTAAGCGGCGGGATGCCATCAAAACTGAATCCGGAGATTAGTTTTAACTACCTGGGGCAGTTTGACCAAGATTTGCAGCAGCATAGGGTTCAATTATCTTCTTACTCCTGCGGTTCAGACTCAAGCGGGAATCAAGCCAGACCATATGTATTGAATATAAACGGAATGATAACCGACGGCCGGCTGACGCTTACGATCAGCTACAGCAGCAAACAGTACGCAAGAGAAACGATCAAGCGGTTAGCGGAAACGATTCAAAGCTGCCTGCGGACAATTATTACGCATTGCGTGCAGAAAGAACAATCGGAACTGACGCCAAGCGATATTTTGTTAAAAGGCATGTCCATTGACGAATTGGATCAGCTCCTCATTCAACTGCCGCATGCAGGTGAGATTGAAAACGTTTATCCGCTCACTCCGATGCAGAAAGGAATGCTCTTCCACAGCTTGCTGGATGAGGACTCGCATTCTTATTTCGAACAAGCATCGTTCGATTTACAGGGAGAGTTAAAGATTGATTGGTTTAAAGCAAGCCTGGAACGATTATTTGAAAAATACGCCGTGCTCAGAACCCGTTTTTACAGCGGCTGGAACGATACTCCTTTACAAATTGTCTATAAAACGCAAAAACCGCAGATTCATTTTGCAGATTTGCGCGACAAAGAGGAGTACCACCGCGAGGATGAAATCGCAGCCTATCAAAGAGAAGACAAGGAAAAAGGATTTGACTTGGCTCGGGGTCCGCTGATGCGCATAGCGATTTTCCGTATGGAAGACCGCAAATATCATTTGATTTGGAGCTTTCATCATATTGTTATGGACGGCTGGTGCCTGTCTCTTATCACGAAGGAAGTGTTTGAGCATTATAGAGCCTTGCAAGAAGGCAGGGAAACAGACCTTTCATCTGCAGCTCCTTACAGCGACTATATCGAATGGCTTGACCTACAAGATCACAGAGAGGCCAAACGGTACTGGAGCGAGTATCTTGACGGTTATAAAGGTGAAACCAGGCTTCTTCATAAAAGAACACAGCATGAACAAAAAGACTATGCCTATGCCAATGTGATCTGCGAATTAGACCGAGAACAGACAAAACGGCTGCAGCAGATCTCAAATCAGCATCAAGTTACATTACATTCACTGATCCAAACGGTGTGGGGAATTTTATTACAGAAATACAGCGGTTCCCCAGACGTTGTCTTTGGCAGCGTCGTGTCCGGAAGACCGGCGGAAATTCCTGGGGTGGAACAGATGATCGGCCTGTTCATCAATACCATTCCGGTCCGCATCCGCTGTGATGAGGACAGCTCTTTTACCGACACGATGCAAATGGTTCAACAAAAGGCATTGGCTTCCCAAGCATACGATACCTATCCTTTATATGAAATTCAGACACAAACGGATCAAAAGCAAAACCTGATTGACCATATCATGATCTTTGAAAACTATCCGATAGGGGAGCAGGTCGAAGAGGCTGGCCATCATGATACAGAGCTGAACATCACGAACTTTCATATGCAGGAACACTCCCACTATGATTTGAATGTGGTTGTCATTCCTGGCGAACAGCTGGCCGTTCATTTCGGTTTTAACGAAAACGAATATGAAAAATCTGATGTGGAGCGGCTTCGCGGACATTTTGAACAGCTCATGCAGCAAGTATTGCAGCAGCCGTCTGTCAAAATAGAAGATTTGGAGCTTCTCTCTCAGCAAGAAAAAGAACAACTGCTCAGCGATTTTCAATCTGGCGGCATGCAATACTCCCTTGAACAAACGATTCATGAGATGTTCGAGGAACAGGCGCACCGTACGCCGAACCAAGCGGCCGTTGTATACGAGGGTAAGCAACTAACCTACGAAGAATTAAACAGGCGTGCCAATCAGCTTGCGCGAACCTTGCAGGCGAAAGGTGTGCGGGCGGATCAGCTTGTCGGCATTATGACTGAACGCTCGCTGGAGATGATTGTAGGCATCTTAGGAGTGTTAAAAGCGGGCGGCGCATATCTGCCGATAGATCCCGATTCTCCGCCTGAACGCATCCGTTATATTTTAAGTGATTCAGGCATCAGCGTATTGCTTTATCGCGGAAAACTGCAGGATAACATCGGCTGCCCGCGGACATGTATCGATCTCTTGGAGGAGCATGCCTGCCACGAAGAAGGAAACGACCTTGCGCTCTCTCATCAGTCGACTCAACTGGCCTATGCCATTTATACTTCCGGCACGACTGGAAAGCCTAAAGGCACGTTAATTGAACATCGCCAAGTCATTCACCTGATTGAAGGGCTGAGCCGCCAAGTTTACTCTGCTCATAAGGGGGAGCTGAATGTAGCCATGCTCGCTCCCTATTACTTCGATGCTTCTGTTCAGCAAATATATGCTTCTTTGCTGTTAGGACATACCTTGTTTATTGTTCCGAAAGAGGTCGTGTCAGACGGTACTGCATTATGTCGTTACTATCGGGAGCATTGCATTGATATTACCGACGGAACACCTGCCCATTTAAAGCTCTTAATAGCTGCAGGTGATTTGCAGGGAGTCCCGCTTCAACACCTTTTAATCGGCGGAGAGGCTCTTTCCAAGATGACGGTAAATAAATTCATGCGATTATTCGGCGACCATGGCGCTGCGCCAAGGATTACCAATGTATATGGGCCTACAGAAACTTGCGTAGACGCGTCCCTGTTCCACATTGAATGTTCTGCAGATGCTTGGATACGCAGTCAAATTCATGTCCCTATAGGCAAGCCGTTAGGCAGCAACCGCATGTACATTCTTGATTCAAAAAAACGGCTGCAACCGGCGGGCGTCCAAGGCGAGCTGTATATTGCAGGGGACGGTGTCGGGCGGGGTTACTTGAACCTGCCGGAACTAACGAATGAAAAATTTGCTGCCGATCCGTTTGTCCCGACAGACCGAATGTACAGAACCGGTGATCTGGCCCGCCTTCTGCCGGATGGAAATATTGAATTTATCGGCCGGATTGACCATCAGGTGAAAATTCACGGTTTTCGGATTGAACTCGGTGAAATCGAATCGGTGATGCTGACCATTCCTGATATTCAAGAAGCCGCCGTAATCGCCCTTGAAGATGCAGATGAAGAATATTACCTGTGCGGATACTATTCTGCGGACAAGCCAATACAGATCAATGAACTTCGCGAAAATATGGCCCGGCATTTACCGGGATATATGATACCGGCTTATTTTGTGCAGCTGGATCAAATGCCGCTTACGCCAAACGGGAAGCTGAACCGTCAGTTATTACCGGCTCCGGTCAACAAGCGGGACAGGGGCATAGAATATGTGCCGCCGCGGACTTCTGCAGAAATTCAGCTGACGGCAATTTGGCAGGATGTTCTCGGGTTAGAGCAGGTGGGGATTCGAGATAACTTTTTTGATATCGGCGGACACTCCCTGCGTGCAACGGCGCTGCTAGCAAAAATACAAAAGCAAATGCATGTCCAAATTCCTTTGCGGGACGTGTTCCTTTTTCCAACCATTGAACAGCTCGCGCGGATGATCACAGAAACGGAGTCAACCGGATATGCCGCTATTCCTGCAATTGAAAAAAGACCTTATTATCCGGTATCCTCAGCACAAAAACGGTTGTACATTCTCAATTCTCTGGAAGGAGGAGAACTCAGTTATAACATGCTGGGCCTGATGGCTGTCGAAGGAACGATTGATCGTGAAAAGCTGCAACAAGCCTTCCTCACACTGATTCAGCGTCATGAATCTTTGCGTACCGGTTTTAAAATGGCCGGCGGAGAACCTGTCCAGTATGTCTTGGATCACGCAGCGTTCGAAGCAGAGTGGTATCAGGCGGAAGAAAATGATGCGGATCTTTATATCCGCCAATTTATCCGTCCGTTTCACCTCGAGGAGCCGCCGCTGCTTCGCGTGGGGCTGATCGAACTTCAACCAGATCGCGGAATTCTGATGTTTGATATGCATCACATTATTTCTGACGGAACATCCATGAACGTATTGATCAAAGAATTTATCCGGATTTATGAAGGAGAGACATTACCGCCTCTGCGCATCCAGTACAAGGACTACGCAGTATGGCAGACCGGAGAAGCGAGATTAAAGGAGATGCAAAATCAAGAAGCCTATTGGTCAGAGCTGTACAGCGGCGACGTTCCTGTCCTTCATCTGCCGACCGATTATATACGGCCGTCGGCAAGGGGTTTTGCGGGAGCCACGATGCATTTTACGCTGGATAAGCAAAAAAGCGCCGGGTTGAAGCAGCTGGCATCCCAAACCGAATCTACTTTATATATGGTGCTCCTTGCGTCTTATACATTATTGCTTTCAAAATACAGCGGCCAGGAAGACATTATTGTCGGCAGTCCTATAGCGGGAAGACCGCATGCGGATTTGGAACCCATTATCGGAATGTTTGTCAATACACTGGCAATGAGAAATTACCCAGAAAAAGGGAAGACGTTCACTCAGTATCTGTCCGAAGTGAAGGAGAATGCACTAAAAGCCTATGAGCATCAAGATTACCCGTTTGAAGCACTGATCGATCAACTGAATATCGCCAGAGATTTAAGCCGTAATCCGTTGTTCGACACCATGTTTGTGCTTCAAAACACGGAGCAGGAACAGTTAGAGATAAAAGACGTATCTTTTAAACCATATCCAAACGAACATACGATGGCGAAATTTGATTTAACCTTAACGGCTGTGGAAGAAGAGTCGGGTATTCATTTTACGATGGAATACTTGACGTCTCTGTTTAAACCGGAAACGATCGAACGTATGATGGGGCATTTTGTACAGCTGATTGATTCGATTATCAAACAGCCTGAAGCCGAACTGGCTAGGTTAAATATGATGACCAAAAAAGAGAATAGTGAAATTCAGAAACTCTTTAACGATACCGCAGTTGCGGATAAACGAATTCCGACAACGGTTCATCAGCTTTTTGAGCAGCAGGCTGAGCTTAACCCAGACCATGAAGCCGTCCTGTTTGGAAATCAGGCACTGACTTATCGCCAGCTGAATGAACGTTCCAATCAGCTGGCGCGCGTCCTTCAAGATAAAGGTGCACGCACTGATCAGGTGGTGGCCGTTCTCACGGATCGCTCAGCAAATATGATGATCGGCATTTTAGCCATATTAAAGGCCGGCGCAGCATTTCTTCCTATCGATCCGGAACTTCCTGATGAACGGCGGGCGTTTATGCTGAAAGACAGCGGCGCCGGAGTGCTTTTGACTGGTGCGGGCCACACCATTCCGCCGCTCTTTGAAGGGGAAGTGCTTTTGCTTGACGATCCGTTATTATATCAAGGCGAAGCGGACAATCTTAATCTTTCCTATTCGGAGAATGATGTAATGTACATCATTTACACGTCAGGCACAACAGGGAAACCAAAAGGAGTTCAGCTGGAGCACAAGACCATGACGAATCTGCTCGCTTATGAAGAGGACCATACGCAGCTGCGCTTTGACAGGGTGCTGCAATTTGCTGCAATGAGCTTTGATGTTTGCTATCAAGAAATCTTCTCCGCGCTTTCAAGCGGAGGAACACTCTATATCATCAGCAATGAGGCAAAACGGGACATACGCCAGCTCAACGATTTTGTCAAAATGCACAGAATCCAAACCGCATTTCTTCCGACTGCGTTCCTGAAGCTCCTTGCCTCGGAGAAACACTATTTCGAACCGTTTGCCGAGTGCGTGGATCACATCATCGCTGCGGGAGAACAGCTCATCGTAACAAGGATGCTGCGAGACATGCTGGTACGCCACCATGTTACATTGCATAATCATTACGGTCCGTCGGAGACGCATGTCGTGACCATGTATACCGTTAACCCGGATACAGATCAGGAACTTCAGCCGATCGGCAAACCGATTTCAAATACGGAGATTTTCATTTTGAATGAATCGGGAACCCTTCAGCCTGTCGGAATCGTCGGTGAACTTTGTATCTCAGGAGTCAGCTTAGCCCGCGGTTATCATAACAGAGAGCCTTTGACGCTCGAAGCATTTGTTCCGCACCCATATGACAAGAAGCAACGGATGTATAAAACAGGCGATCTGGCCCGTTACCTTCCCGACGGAAATATTGAATATGCGGGGAGGATGGACCATCAAGTCAAAATCCGCGGTTACCGGATCGAACTCGGCGAGGTGGAGGCCGCTCTTCTCGAGCCTGTACAGGAAGCAGTTGTTCTGGCAAGAGAAAATGCAGATGGGCAAAGCGATTTGTATGCCTACTTTACAATGGAGCAATCACTGCCGATCAGTCAGCTGAAAGAAAAACTTGCTGATCAAATACCGAGCTACATGATCCCTTCATACTTCATCCAACTGGAGAAACTGCCGTTAACCTCCAATGGAAAAGTCAATCGAAGAGCATTACCGATGCCTGAAGCCGGTTTGCAAACCGGAATCGACTACGTTGCTCCGCGGACGAGTATGGAAGAGCAGCTGGTTTGCATCTGGCAAGACGTTTTGAAAGTAAAAGAAATTGGCGTAAAAGATAACTTTTTCGATCTGGGCGGCCACTCGTTACGGGGGATGACGCTCATCGCCAAAATTCATAAGCAATTCAGCAAAAACATTTCATTAAGAGAGGTATTTCAATGTCCGACCATTGAAGAAATGGCAAAGGCTATCGCAGAAGCTGAAGCCGACAGGCCGGATTACATTCCGGCAGCCGAGGAAAAAGATGTCTATCCTGTATCCTCCGTGCAGAAGATGGTTTACCTGTCAACACAAATTGAAGGCGGCGAACTTAGCTACAACATGCCGGGAATCCTTACATTAGAAGGAAAAATCGACATGAATCGCTTGCAAACCGCTTTTCAAAGACTGATTCAGCGTCATGAATCACTGCGTACCGGATTTGAAATGATCCGCGGGGAACTGATGCAGGTGATCAAGCCGGAGATAAATTTCTCTATAGAAAGGGACAAGGCAGCATCGGACGAGGTTGAGGAACTTTTCCGCGCCTTCGTGCGGCCGTTCGATCTCAGCCGGGCTCCGTTACTTCGGGCCGGACTCATCGAACTAGAACAAGACCGGCAAATTTTTATGTTTGACATGCACCACATCATTACGGACGGAGCATCCATGAATATTTTTGTCGAAGAACTGATTCAATTGTATGACGGCAAAGAATTAGCTCCGCTCCGTATTCAATACAAAGACTTTACTGAATGGAAACACCAAAAAGAACAAAGAGAACGAATCAAGAGGCAGGAAGAGTATTGGCTCGGTGTATTTGAAGAAGAGCTGCCGACATTCGAGCTGCCGAAGGATTTTGCAAGACCGCCGGTGAGAAGCTTCGAAGGGAAGCGGCACAATTTTACGCTCGATAAGACTGTCATCGAAGGTATAAAACAGCTGGAAGAATTGACAGGTACAACCGCTTATATGATTTTGTTTTCGGCTTACTCCATACTGCTGGCTAAATACAGCGGTGAGGATGATATCGTCGTCGGCACACCTATTGCGGGAAGAATGCATGAAGATTTACAGCATATCATCGGAATGTTTGTCAATACATTAGCCATCCGTACCGCTCCAATAGCGGAGAAGACGTTTTTGGATTATATAACGGAAACGAAAGAAACAATGCTGAAGGCTTATGAACATCAAGAGTATCCTTTTGAAGAATTAGTTGAGAAATTGGGAGTGAAACGTGATTTAAGCCGCAATCCGTTATTCGATACGATGTTCGTACTCCAAAATACGGAACAAACGGATATTGAGGTGGACTCTCTCGCTGTCCGACCTTATGAACAAACGGAGACGGCTGCCAAGTTTGATTTACAGCTGACCTTCGTAATGGAACAAGATGAGATACAAGGCAGCTTTGATTACTGCACGAAGCTATTTAAAAAGAATACCATTGCTGTATTGGCCAAGGATTACATCATGATCCTTTCGGCGATTTTACGAAACCCATCCATTCCTTTAAAAGAGATTCAATTAAGTGAAAAAGTAAAGAAAAGTAAACATCTCGCAAGCACAATCGAATTGGATTTTTAATCACAGGGGCAGCGGCTGTCCCGGCACGAATGCCGCGGCTGCCGCCAGCCGCCTCAAAACAGGAATGAAACCAACTGGAGGGATATGAATGTCAGAATTCAAACAACAGGAGTTGTTCTGGGGCCGAATGTTTCATGCGGAAGACCGCCCCAGTGCATTCCCTTCGTTTCAAGTGTCCGACTCAACAGTAAAGCGTGATGTAACGGGTGCATCTGATTGTATTCACAGTACATTGAGTGCGGATGTATCACGGCGCATCCTGACCATGACGCATCAAACGCCAATGGCCGTCTATCTGGTTCTTCTGATCGGTATTGAATCCCTATTGTATAAATACACAGGGGAAGAGGACGTGATTACAGGAGTTCCAACTTTTGAAGACGAAACTGATGAAGATCTTCGTGCAGATCAGCTCATGTTAATTAAACAGCATATCAATGCCGACAGTACGTTTAAATCGATATTCCATGAACTGAAACATACGCTGGATGAAGAGATCTTATATCAGGACGTTCCCTTTGATAAAATGGCCGGACAGCTGCAGCTGAACTACAATGAAAATCATTTGCCGAACGTCCATACAGTTGTTTCGTTAGAGCAAATTCATCCTGATCGTTTCATAGAAACGGCTGCTTCAGATGCTTTGTTTCAGTTTGCTATGGGAGAAGACTCGATTGATGTAAAGTTATTTTTTAATGAACAAGTCTATGACCGTCAGTATATGATGCAGGTGCTCGGGCATTTAAACCGCCTGTTTTCCGTCATATTATTTCAGCCCGATCTCACCCTCGGCGAAGTGGATATTTTGTCAGAAACGGAGACAAATACATTTCTCGTTGACTATCATCATACGAAAACTGAATATCCGCGAGATAAGACGATTTATCAGTTATTCGAAGAACAGGTGAAACGGACACCGGATCAAGCAGCCATTATTTGCGGAGATAGGCAATTCACGTATCGCCAGCTCAATGAACGTGCCAATCAATTAGCCCGAACGTTAAGGGCCAAGGGCGTAATGGCGGACCGGCTTACCGCCATCATCAGCGAACACGAGATTGAACTGGTTGTGGGAATACTGGCCGTTTTAAAAGCGGGCGGAGCCTACGTGCCGATTGATCCGGATTATCCGAAGCATCGCATACAGTATATAGTAGATGACTCCCAAGCTGATATCGTCTTAACGCAGAGCCATCTCTCAAAACAATTGGAACTTGCAGGCACAATGGTTTTCATCGATGAGGAAAGCTCTTACCACGAAGACGGATCTGACCTTGAACCGATCAGCAGTACAAAGGATTTGGCCTATGTCATTTACACGTCAGGTTCCACAGGCAAGCCGAAAGGTGCAGCAATTGAGCATCAGGGTTTAACCAATTATATTTGGTGGGCCCGCGGGGTATATGTCAAAGGAGAAAAAACCAATTTTCCGCTATATTCGTCCATCGCTTTTGATCTGACGGTAACGTCTGTTTTTACTCCGCTTATTACCGGAAATACCATCATTGTCTACGGAGGAGAAAACAAGGCGGTCTTGCTTGATTCCATTATTCAGGATTCAAGGGTGGATATAATCAAGCTGACCCCGGCTCATTTGCAATTATTGAATGAAATGAACATCTCTCCTGAGTGCACCATTCGGAAATTCATTGTTGGCGGCGATAATTTAAGCACGCGTCTCGCCCGAAACATCACCGGGAAATTCGGCGGCAGAATCGAAATCTTTAACGAATACGGTCCTACAGAGACGGTCGTCGGCTGTATGATTTATTCCTTTGACCCGCAAAACGACAGACGGGAATCAGTGCCGATCGGAACCGCCGCGGACAACATGAACATTTATGTGCTGGATAAGAGCATGAAACCGGTGCCGATCGGCGTCCCGGGGGAAATGTATATCAGCGGGGACGGCATCGCCAGAGGATATTTGAACCGGCCGGAATTAACATCTGAAAGGTTTATGCGGAACCCCTTTGTACCGGAGGCGAAAATGTACCGGACGGGAGACTTAGCAAGATGGCTGCCGGACGGTAATATCGAGTACCTCGGAAGGATCGACCATCAGGTAAAAATCCGCGGGTACCGCATTGAAATCGGGGAAGTCGAAGCGGCATTTTATCAGATACCGCCGATTCAAGAAGCTCTCGTTATCGCACAAGAAACCAATGGGGAAACGACTTTGTGCGCCTATTATACCGCCCAGCATTCTTTAACCGCGGGTGAGATCCGTGAACATCTGTCCCGGCAGCTTCCGGCCTATATGATCCCTGCTTATTTCGTACAGCTGACAGCGATGCCGCTTACTTCCAACGGAAAAATAGATCGTCAGGCGCTGCCTGCTCCAACGGGAAATCTCACTGGCAATACCTATACAGCTCCGCGGACGGAACTGGAAAAAATACTAGCCGGGGTGTGGGAATCGGTATTAGGCACAGAACAGGTCGGTATCGACGATCATTTCTTTGAGCTGGGGGGAGATTCGATTAAGTCGATTCAAGTGACCTCAAGCCTGTATCAAGCCGGATATAAGCTTGATATCAAACACTTGTTCAAACACCCGACAATCTCCAGACTTGCTCCTTTTGCAGAGCCGGTCACCCGGATCGCAGAACAAGGGGAAATGAAAGGCCAGGCTTTACTGACACCGGTTCAGCATTGGTTTTTCGCCCGGCAATACCCTGATCCGCACCATTATAATCAAGCCGTCATGCTTTATTTTAAAGAGGGTTTGGTTGAATCGAAGCTTCGGGAAGTCATGAAGAAGATTGCAGAACATCATGATGCTTTGCGCATGGTCTTTGTTCCGGCAGATCATGGATACCAAGCCCGAAACCGGGGGATCGGTGAAGGAGATTTATTCAGCCTCGAAGTTATCTCTTTACTCGAAGAAAACAACCCTGCCCAAACGATTGAAACGATATCCGATGATATTCAGCAATCCATCCATTTAGCCGAAGGACCGCTCATGAAATTGGGATTGTTTCAATGTCAGGATGGAGATCATTTGCTGATTGCGATTCATCATCTGGTCGTCGACGGCGTATCGTGGAGAATTTTACTTGAAGATATAACGTCAGCCTATGGGCAGCTCCAAAACGGCGAAGCGATCAGGCTTCCTAAGAAGACGGATTCCTACCTGCTATGGGCTGAACGATTGAAACGTTATGCAGAAAGTGCAGAATGTGAAGCGGAAAATCAATACTGGTTCGGGCAGAGCCATATCCCGCAATACAAATTGCCAAAGGACAATGAGCAGGAGGCCGGTCTGGCCGAAGACCGGGAAACCATCGTCGTCCAATGGACAGAGGAAGAAACCGAATGTTTATTAAAAAGATCGAACCGGGCCTATACGACTGACATTAACGATTTATTGCTGACCGGACTCGGTACAGCTGTTCATCGCTGGACTGGACATGAAGAGATTCATGTTCATCTGGAAGGACACGGCAGGGAATCTATTTTTCAAGACCTTGATATTTCACGTACGGTGGGCTGGTTTACAAGCCAATACCCGGTTTCTCTTCAAATTCAGGCAGATCAAGACGTCTCACAGCGGATTAGGACGGTGAAAGAACAGCTGCGTCAAATTCCCCAAAAGGGAATAGGATACGGCCTGATTAAATATTTGTCCGATCATCCGAAGGCATCTGAATGGACCGGACATCCGGAAATCAGCTTTAATTACCTCGGTCAATTTGATCAAGATCTCGAAAATGGAGGGATCGAGGTATCTCCTTACTCCGGCGGGAAAATTGCGAGTGACAGGCATCCCATTACCTATACGCTTGACATCAACGGCATGATTTCAGACAGCCGGCTGTCATTGGCCATCAGTTACTGCGGTAAACAATATCGAAGAGAAACAATGGAAATATGTGCCGATCTCTTAAAGAGCAGCCTGAAAGAAGTCATCGAACATTGTGCAGTTCAAGAACAAATTCAGCTGACGCCGAGCGATATTTCGCTGAAAGGCATCACAATCGCGCAATTAGATCAATTTGTTCAGCAAACACAGCATATCGGAGAGATTGAAAATATATATCCCTTAACACCCATGCAAAAGGGCATGCTGTTCCACAGCTTAATCGATTCGGCTTCCAGAGCTTACTTTGAACAAGCCGCTTTTGATCTCAAAGGCGACTTGGATATCGAGGCATTTACGATGAGTTTGTCACAGCTGGCAGAAAGACATGAGATACTCCGGACTCATTTTTACACCGAATGGAAAGATCAGCCTTTGCAGATCGTATTCCGAAAAAAACCTATCGAAATAACCGTAGAAGATATTCGAAGCATGGAGAATGAAGAACGCGGCGAGTTCATTACCGACTTTGTACAAAAAGATAAGGCAAGAGGATTCGATCTTACCCAAGATGCGTTAATGCGCGTATCAATCCTTCTTACAGAGGATGATCAAGCCCGATTGATATGGAGCTTCCATCATATTTTAATGGATGGCTGGTGTCTGCCGCTTATTACGAAAGAAGTATTTGAAACCTATTATGCGATTCTTGACAGAAGAAGTCCCGAGCGGAAGACCGTCACCCGGTACAGCCGTTATATCGAATGGCTGGAAGACCAGAATCATAAGAATGCTTCAGCCTATTGGCAAACATATTTGAACGGTTATGAAGGGCAAACCGTTTTGCCGAAAGAATCAGTTCCAAACCAAGCTGAAGGATATCAAAAACAAATGCTCGCATTCCCGCTCGGAAAGCAGCTCTCAGAAGAAATCAAACGGACCGCAAGTAAGCACGGCGTCACTGTGAATACCTTCATGCAAAGTGCGTGGGGATTGTTGCTGCAAAGATACAACAACAGTCAGGATGTGGTTTTCGGAACGGTCGTATCCGGCCGCCCGGCGGAAATTCCGGGTATCGAATCCATGGTCGGCTTATTTATCAACACCATTCCCGTACGTATTACTTCACAGTCTGAAATGACCGTGGAAGAAGTGTTGAAATTGAGCCAGGAGCAGGCATTGGCCTCTCAGGCATATGATACTTTTCCGTTGTACGAAATTCAGGCTCAAACCGAACAAAAACAGCAGCTGATCAGTCACATCATGGTGTTTGAAAACTACCCGGTCGAGAAGCAAATGGAGCATATGAAACAGGACCATGACGCGCTGGACATTATCAATTTCCATATGGAAGAGCATACCCATTATGATTTCAATTTCATTGTCATGCCGGCGGGGGAAATTGACATTCATTTTGTCTACAATGGCAATGTCTATGATCATGCAAGCGTCAAGCGGATGGAAGAGCATTTGATGCAAATCATTAAGCAAATGGTCAACAATCAGGCGGCCCGCGTCCAAGACCTGAATATACTTACGGCGGACGAACACTCGCTCCTCATAGAGGCATTTAATGATACGGCCGCAGATTATCCAAAGGAAAAGACGCTTCATCAATTGTTTGAGGAACAAGCTGAGCGAACGCCTGAGCAAACGGCTGTCGTATACGGAAAAAGCCGGCTGACTTACCGGGAACTGAATGAAAGGGCGAATCAACTTGCCCGGACATTGCAATCAGAAGGCGTGCAGCCAGATCAGCCGGTCGGAATGATGGTCGAACGTTCACTGGAAATGATCATCGGAATCTTTGGAATCCTAAAAGCCGGCGGTGCGTATGTACCGATCGACCCGAGTTATCCGGACGGCCGGATTCAGTATATCTTGGAGGATTCAGGCACGAAGCTGCTGTTAGTACAGAGTCATTTGAGGGAAGGCTTACCTTTTAGAGGAAAAGTGCTTGATCTGGAAGATCCGCGATTCTCCGGGGAAGATGCTTCAAACCTGGAACAGACGGCAGGTCCGAATCACTTAGCTTATGTCATCTATACATCGGGATCAACAGGCCGACCGAAAGGGGTTATGGTTGAGCATCGATCTGTCATCAATCGTCTCGTTTGGATGCAGGAACACTACCCGCTGGATGAACGGGATGCCATTCTGCAAAAAACCCCAATCACTTTTGATGTATCGGTATGGGAATTGTTCTGGTGGTCGATGACGGGTTCAAAAGCGGTATTGCTGCCAAACGGAGGAGAAAAGAACCCTGACGTCATTTTAGATACGATTGCGCAGAAAGATATCACCGTTATGCATTTTGTACCTGCGATGCTGCACGCTTTTCTTGAATCCATGGAGCAGAAATCCTCCGGGGAATTAAAAAGAAAGCTTGAAACATTAAGATATGTATTTGCAAGCGGCGAGGCTTTGACGCCTGCGCACGTTTCCGGGTTTCACCGTTTGATCACACCGGCAGGCAAGGCTCAAATCATCAATTTATACGGACCGACCGAAGCGACGATCGATGTATCGTATTTTGAATGCGAAGAGGGAGACAAGCATGCCTCAGTTCCGATAGGCAGACCGATTTCCAACATTCAATTGTATATCGTACAGCCCGAATCAGAACATCTTCAGCCGCTTGGTGTGGCAGGAGAGCTGTGTATCGCCGGAGACGGGCTCGCGCGGGGATATTTGAACCGTCCGGAGCTGACAGCAGAGAAGTTTGCAGATCACCCGCTTGAAGCAGGAAAGCGGATGTACCGGACAGGAGACTTAGCAAGATGGCTGCCCGACGGCAATATTGAGTATTTGGGGAGAATTGACCATCAGGTGAAAATCCGGGGCTACAGAATTGAACTCGGCGAGATTGAAACAGCTTTATTGCAACTGGAATCTGTCAAAGAAGCGATCGTCATCGCGATTGAAGAAGAGCGCTCCAAGCAGCTGTGCGCCTACTTGACAGGGGACGAAGCATTCAATACGGCGCAGCTGAAGCGGCATTTGCTGAACAAACTGCCCGCGTATATGATTCCGGCCTATTTTGTACAAGTAGAGAAGATGCCGATAACGGCTAACGGCAAAATTGACCGAAAAGCTTTGCCGGCTCCTGAAGAAAATAGAGTGACCGGAACTGAATACGCAGCACCGGGAACGCTGATTGAAAAGCAGCTGGCCGATATATGGAAAGATATTTTAGCGCTTCCTGAACCGGGGATTAAAGATGACTTCTTTGACGCCGGCGGCCATTCACTCAAAGTATTGCAGCTCATTCATCAAATTAACGCCAGTATGGGAATCAACATGCATTATCAAGCCGTATACGATTTTCCTACCATCGAAACAATGGCACGCGCCATTCAAGCGGCAGTTTTTGAATCCAAGACGGATAACGTATTCGTCAAGCTGAATCAGGAAGGTTCAATCCCTGTGTTCTGTTTCCCGCCTTTAATCGGGTACGGGCTGGTTTATAATGAAATGGCGAAAAGGCTTGATGGCGAATGCACCGTCTATGCCGCTGATTTCTTAGAAGAGCCGGCTCATGAGAAGGAGATCGTTGCCCGGTATGCAGAAAGCATGATAAACATTCAGGAACAAGGACCTTTTGTTTTGCTTGGATACTCTGCAGGATCAAATTTGGCATTTGAGGTTGCCAAAGCTTTGGAGAATCGGGGACGCACCGTATCTGATGTGCTGATGCTGGATTCTAAAATCACCGATTCGGTAACGCATTTATCGGAAACGGAAATTGAAGAGATCGTCCGTTTGAATATAGATATCATTCCGGACTATTACAGAGAAATATTAACCATACCTTCGATTAAGGATAAAATCAGAAGCTATCTCACGTATCACAATGAACTGATCAATTCCGGCAACGTTAATGCCAATATCCATCATTTATTATGCGGCGATTTGACCGATGATAGAGGCTGGGCGCAATCCGCTGCACAGCATTACCTTGAATACAAATTAAAAGGAGACCATGTGACGATCTTTGAACCTCACAATATCGAAGAAAATACGGATGCCATTCGATCTATTATCAAAAGGATTGAAGAACGGCACCAGCACGGGCTTGTTCTTGAAGAACAACTGTTATTGGGATCGTTTGCGGGAGACACAAAGTTTGACAAAATGTAAATGATCCAGTGCAAAATGCCGCTGGATGACAGTCAGCGGCATTTTGTACGAAAAAGAAGAGAGTAAACTCATAGGCTTGAAACGCTTTCCTCGTTATGCGTTCTCCATGACCATTTGTCATCAAATGTTAAAAGAAAAGCCTCATTTTAAGGAAAAAGAAGGATATTCAGTCATGACAAAAAAGTGCAGCGTATGTTTATTGATTCTTGCTTTATTATTGAGTTGCTTTACTGGGAAGTCGTCGTATGCTGCCAACACTGCAAATTCCCAAAAGGAGCCCTATTTTAGGAAAAGGAGGATATTAAGCAATGAGGAAAAAATGTAACTTATGTTTATGGGTCCTTGCTTTATTATTGAGTTGCTTCACTGGGAAGTCTGCGTATGCTGCCAGCACTCCAATTGCAAAACATGTTGGGAATTC | |
