# Supplementary figures and images for: Bacillus subtilis engineered for topical delivery of an antifungal agent
Source: PLoS One. 2023 Nov 30;18(11):e0293664. doi: 10.1371/journal.pone.0293664 (PMC10688720; doi:10.1371/journal.pone.0293664)

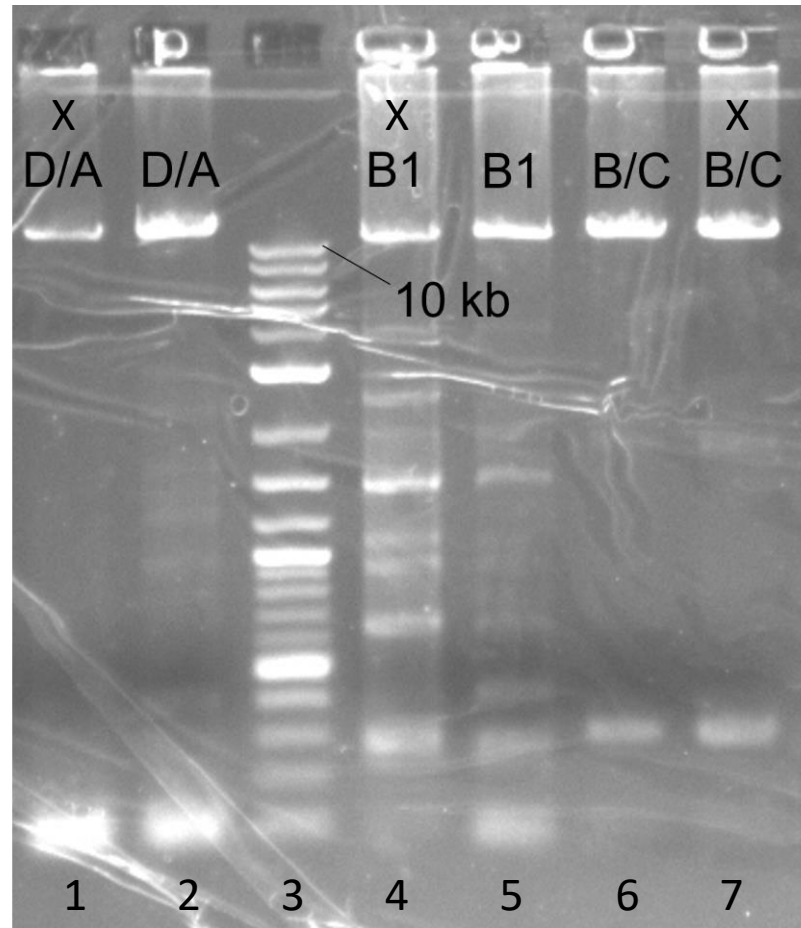

Supplement: S1 Raw image — D/A encompasses a region including ituD and ituA. B1 encompasses part of ituB. B/C encompasses the remaining section of ituB and ituC. Duplicate lanes show replicates of the same PCR reaction. This image was used to generate Fig 1 in the manuscript. “X” marks lanes that were cropped out of the figure. Numbers at the bottom of the image indicate the order in which samples were loaded into gel. The gel was imaged using a UVP UVsolo touch stand-alone gel documentation system. (PDF) [file pone.0293664.s003.pdf]
